# Sdd3 regulates the biofilm formation of *Candida albicans* via the Rho1-PKC-MAPK pathway

**DOI:** 10.1128/mbio.03283-24

**Published:** 2024-12-17

**Authors:** Li Mei Pang, Guisheng Zeng, Eve Wai Ling Chow, Xiaoli Xu, Ning Li, Yee Jiun Kok, Shu Chen Chong, Xuezhi Bi, Jiaxin Gao, Chaminda Jayampath Seneviratne, Yue Wang

**Affiliations:** 1 A*STAR Infectious Diseases Labs (A*STAR ID Labs), Agency for Science, Technology and Research (A*STAR), Singapore, Singapore; 2 Singapore Oral Microbiomics Initiative, National Dental Research Institute Singapore, National Dental Center Singapore, Singapore, Singapore; 3 Bioprocessing Technology Institute, Singapore, Singapore; 4 Duke-NUS Medical School, National University of Singapore, Singapore, Singapore; 5 State Key Laboratory of Mycology, Institute of Microbiology, Beijing, China; 6 Oral Health ACP, Duke NUS Medical School, Singapore, Singapore; 7 School of Dentistry, The University of Queensland, St Lucia, Australia; 8 Department of Biochemistry, Yong Loo Lin School of Medicine, National University of Singapore, Singapore, Singapore; Tel Aviv University, Tel Aviv, Israel

**Keywords:** *C. albicans*, biofilm, *SDD3*, PKC-MAPK pathway, chitin, fungal cell wall

## Abstract

*Candida albicans*, the most frequently isolated fungal pathogen in humans, forms biofilms that enhance resistance to antifungal drugs and host immunity, leading to frequent treatment failure. Understanding the molecular mechanisms governing biofilm formation is crucial for developing anti-biofilm therapies. In this study, we conducted a genetic screen to identify novel genes that regulate biofilm formation in *C. albicans*. One identified gene is *ORF19.6693*, a homolog of the *Saccharomyces cerevisiae SDD3* gene. The *sdd3*∆/∆ mutant exhibited severe defects in biofilm formation and significantly reduced chitin content in the cell wall. Overexpression of the constitutively active version of the Rho1 GTPase Rho1^G18V^, an upstream activator of the protein kinase C (PKC)-mitogen-activated protein kinase (MAPK) cell-wall integrity pathway, rescued these defects. Affinity purification, mass spectrometry, and co-immunoprecipitation revealed Sdd3’s physical interaction with Bem2, the GTPase-activating protein of Rho1. Deletion of *SDD3* significantly reduced the amount of the active GTP-bound form of Rho1, thereby diminishing PKC-MAPK signaling and downregulating chitin synthase genes *CHS2* and *CHS8*. Taken together, our studies identify a new biofilm regulator, Sdd3, in *C. albicans* that modulates Rho1 activity through its inhibitory interaction with Bem2, thereby regulating the PKC-MAPK pathway to control chitin biosynthesis, which is critical for biofilm formation. As an upstream component of the pathway and lacking a homolog in mammals, Sdd3 has the potential to serve as an antifungal target for biofilm infections.

**IMPORTANCE:**

The human fungal pathogen *Candida albicans* is categorized as a critical priority pathogen on the World Health Organization’s Fungal Priority Pathogens List. A key virulence attribute of this pathogen is its ability to form biofilms on the surfaces of indwelling medical devices. Fungal cells in biofilms are highly resistant to antifungal drugs and host immunity, leading to treatment failure. This study conducted a genetic screen to discover novel genes that regulate biofilm formation. We found that deletion of the *SDD3* gene caused severe biofilm defects. Sdd3 negatively regulates the Rho1 GTPase, an upstream activator of the protein kinase C-mitogen-activated protein kinase pathway, through direct interaction with Bem2, the GTPase-activating protein of Rho1, resulting in a significant decrease in chitin content in the fungal cell wall. This chitin synthesis defect leads to biofilm formation failure. Given its essential role in biofilm formation, Sdd3 could serve as an antifungal target for biofilm infections.

## INTRODUCTION

Myriads of microorganisms live in the human body as commensals, normally not causing harm to the host. Among these is the fungus *Candida albicans*, commonly found in the oral cavity, gastrointestinal tract, genitourinary tract, and skin of most healthy individuals (1–4). However, *C. albicans* can cause severe infections under certain circumstances, especially when the host microbiome is perturbed, normal tissue barriers are damaged, or immune defenses become compromised (5). For instance, vulnerable population groups, such as individuals with HIV/AIDS, transplant recipients, and chemotherapy patients, are especially susceptible to *Candida* infection (6). *C. albicans* remains the most common cause of fungal infections, ranging from mild superficial infections to life-threatening candidemia, with mortality rates up to 47% (7, 8). Recently, the World Health Organization categorized *C. albicans* as a critical priority pathogen on its Fungal Priority Pathogens List.

*C. albicans* is a polymorphic organism and may exist as round yeast cells, tubular hyphae, and dumbbell-shaped pseudohyphae (9). In a pathogenic state, *C. albicans* cells adhere to and invade host cells, secrete hydrolytic enzymes and toxins to damage host cells, undergo morphological transitions, escape the recognition by the host immune system, and can even form biofilms (10). The *C. albicans* biofilm is a spatially organized three-dimensional structure composed of yeasts, pseudohyphae, and hyphae, typically embedded within a gel-like protective substance called extracellular matrix (ECM) (11). *C. albicans* can form biofilms on both biotic and abiotic surfaces, such as catheters and dental implants, conferring high resistance to most classes of antifungals and host immunity (5, 12). Biofilm formation is a complex and sequential process consisting of four successive phases (5, 11–13). Adherence is the first step, during which yeast cells stick to a surface and proliferate to form a basal layer that anchors the biofilm to the substrate. In the next initiation step, some adherent yeast cells switch to pseudohyphal and hyphal growth. Thereafter, in the maturation step, *C. albicans* continues to proliferate and elongate, constructing a dense network of yeast cells, pseudohyphae, and hyphae. Meanwhile, an abundant ECM, composed of carbohydrates, lipids, proteins, and DNA, is synthesized. This ECM encases the fungal cells, forming a protective physical barrier. Finally, in the dispersion step, non-adhering yeast cells detach themselves from the mature biofilm, enter the bloodstream, and disseminate to establish new sites of infection (14, 15).

The complex biological process of biofilm formation requires the coordinated activities of multiple signaling pathways and gene regulation networks (16). For example, a master transcriptional regulatory network consisting of six major transcription factors, Efg1, Rob1, Ndt80, Tec1, Bcr1, and Brg1, has been shown to control the normal process of biofilm formation (17, 18). These transcription factors regulate each other’s expression, and a small change in the expression of any one of them can significantly affect the biofilm-forming capability of *C. albicans*. Subsequently, three additional transcription regulators, Rfx2, Gal4, and Flo8, were identified, forming an expanded regulatory network with the six master transcription factors to temporally regulate the biofilm formation (19). Whole-genome transcriptomic analyses and chromatin immunoprecipitation experiments revealed that these transcription regulators form a complex and interconnected network, in which the individual regulators control their own expression, each other’s expression, and the expression of ~1,000 other target genes (17). Furthermore, the promoters of most of the target genes are bound by two or more transcription regulators (17). Compared with the transcriptional regulatory network, upstream signaling pathways involved in biofilm formation have been less studied. The Ras signaling pathway has been shown to regulate biofilm formation, as well as some other pathogenic traits such as cell adhesion, filamentation, and white-to-opaque switching (20). Ras regulates the cAMP-protein kinase A (PKA) pathway, which controls the expression of a specific repertoire of genes required for hyphal morphogenesis and biofilm development (17, 20–22). Mkc1, the mitogen-activated protein kinase (MAPK) of the cell wall integrity pathway (23, 24), has also been shown to be required for normal biofilm development (25). However, how this pathway regulates biofilm formation remains unclear.

The Mkc1-mediated cell integrity pathway is primarily involved in cell wall biogenesis (23, 24). The *C. albicans* cell wall maintains the cell morphology and osmotic stability, provides protection against environmental stresses, and supplies adhesin proteins during the adhesion stage of biofilm formation (26). The fungal cell wall is a two-layered structure, with the inner layer consisting of chitin, β-1,3-glucan, and β-1,6-glucan, and the outer layer consisting of mannosylated glycoproteins (26). Chitin comprises about 1%–2% of the yeast cell wall dry weight and is essential for cell wall integrity (27). *C. albicans* chitin is synthesized by four chitin synthases: Chs1, Chs2, Chs3, and Chs8 (28). Chitin synthesis and *CHS* gene expression are coordinated by the protein kinase C (PKC)-Mkc1 MAPK (hereafter referred to as PKC-MAPK), high osmolarity glycerol-MAPK, and calcium-calcineurin signaling pathways (29). The PKC-MAPK pathway (i.e., the cell wall integrity pathway) is activated by the Rho1 GTPase to upregulate chitin synthesis in response to cell wall stresses (29). Although the construction of the fungal cell wall is well understood, the involvement of major cell wall components in biofilm development remains nebulous (30). So far, only a few cell wall proteins, such as Als1, Sap6, Rbt1, and Pga59, have been implicated in cell adhesion and/or biofilm formation (31–34).

Biofilm formation is one of the major causes of antifungal resistance. Many intrinsic properties of biofilm, such as high cell density within the biofilm, the dense complex structure of the ECM, the existence of persister cells, and the expression of antifungal resistance genes, all contribute to its antifungal resistance (35). Due to its natural resistance to existing antifungals, the biofilm lifestyle of *C. albicans* presents a severe challenge to treatment and is one of the leading causes of candidiasis-mediated mortality. Therefore, there is an urgent need to develop novel therapeutic strategies effective against biofilms. Identifying new targets of antifungals that can penetrate or disrupt biofilm may provide promising therapeutic approaches. Components of the transcriptional networks and signaling pathways responsible for biofilm formation could serve as potential targets for antifungal development. Identifying novel genes/proteins essential for biofilm development may help expand the scope of antifungal targets.

In this study, we conducted a genetic screen aimed at discovering novel genes that govern the biofilm formation of *C. albicans*. One of the identified genes, *SDD3* (*ORF19.6693*), encodes a putative metalloprotease and is required for forming normal biofilms. We found that Sdd3 physically interacts with Bem2, the GTPase-activating protein (GAP) of Rho1, inhibiting Bem2’s GAP activity to regulate the level of active Rho1. Depletion of Sdd3 downregulates Rho1 signaling and its downstream PKC-MAPK pathway, resulting in the loss of chitin content and the failure of proper biofilm formation. Collectively, our studies reveal that chitin plays an essential role in biofilm formation and identifies Sdd3 as a potential antifungal target to treat biofilm-associated infections.

## RESULTS

### A genetic screen identifies novel regulators of biofilm formation

**Fig 1:**
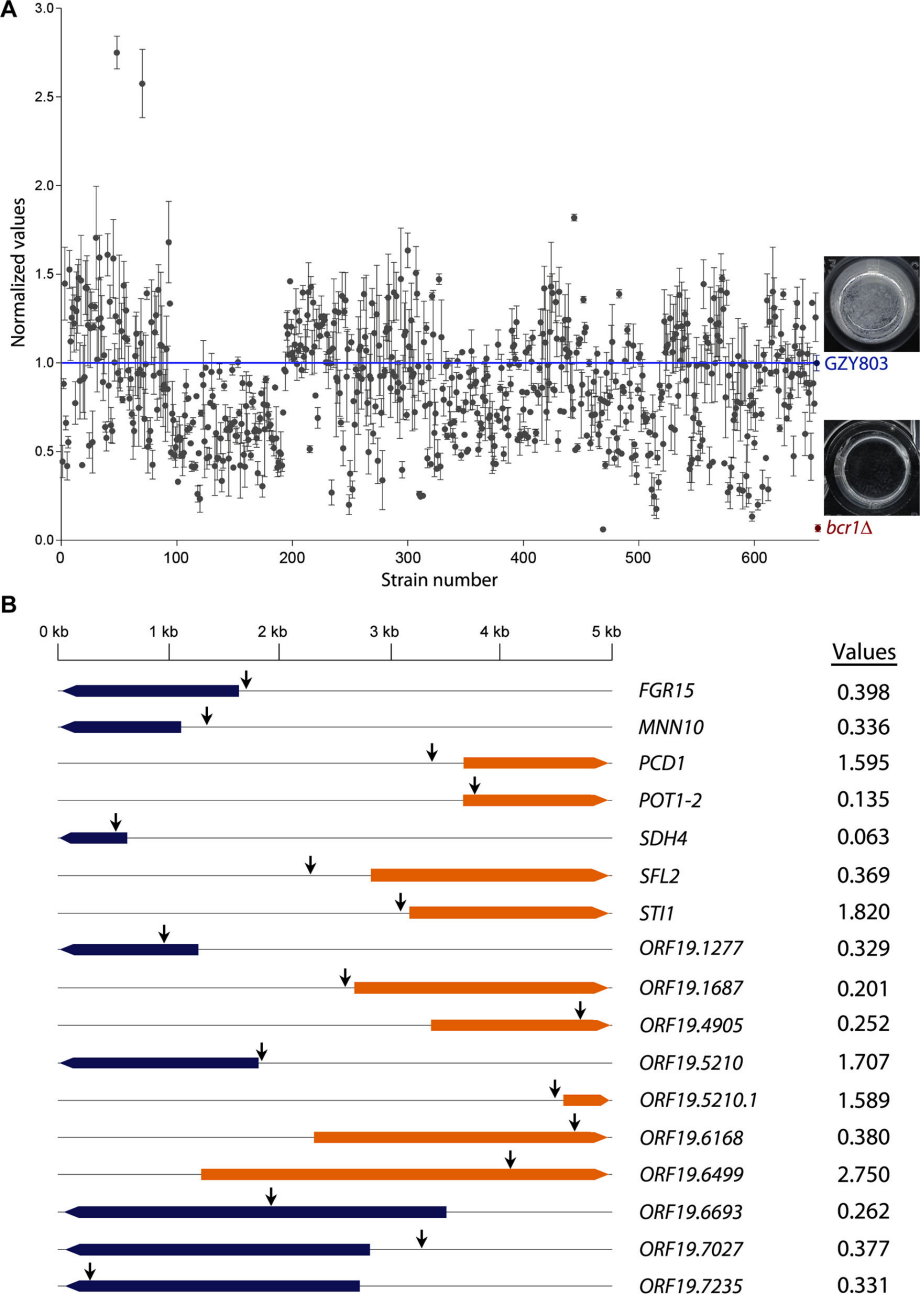
Screening of *C. albicans* haploid mutants identified novel regulators of biofilm formation. (**A**) Comparison of biofilms by measuring OD_600_. Biofilms formed by various haploid mutants were measured at OD_600_ and normalized against the WT strain GZY803. Mutants with normalized values <0.4 or >1.5 are considered to have defective or enhanced biofilm formation, respectively. Data shown are the average of three independent experiments, and error bars represent standard error mean (SEM). The known biofilm-defective mutant *bcr1*∆ (GZY1095) was used as a negative control, and the microscopic images of the biofilms formed by GZY803 and *bcr1*∆ are shown. (**B**) Identification of genes mutated in the haploid mutants with abnormal biofilm formation. The transposon insertion sites (indicated by arrows) were identified by inverse PCR and DNA sequencing. These gene mutations resulted in either defective (values < 0.4) or enhanced (values > 1.5) biofilm formation.

Using the recently developed *PiggyBac* transposon-mediated mutagenesis technology (36, 37), we generated haploid *C. albicans* mutant libraries with transposons randomly inserted into different loci spanning the entire genome. To identify novel biofilm regulators, we screened approximately 650 independent mutants in biofilm assays in 96-well microtiter plates. The wild-type (WT) parental haploid strain (GZY803) and the known biofilm-defective *bcr1*Δ mutant (GZY1095) (38) were included as the positive and negative controls, respectively. The biofilm assays were conducted over 72 h, and the formed biofilms were assessed by measuring optical density (OD_600_) and normalized against that of the WT strain GZY803. While most haploid mutants had a normalized value close to 1.0, seven mutants exhibited normalized values higher than 1.5, indicating enhanced biofilm formation. On the other hand, 27 mutants showed normalized values lower than 0.4, indicating biofilm formation defects (Fig. 1A).

Next, we identified the genes affected by transposon insertion in the 34 haploid mutants using inverse PCR and DNA sequencing, as described previously (36, 37). Because some mutants contained transposon insertion in identical places or in different locations within the same open reading frame (ORF), we ultimately identified 17 genes in total. Eight of these genes had transposon insertion within ORFs, while the remaining genes had transposon insertions in the promoter regions (Fig. 1B). The putative or known biological functions of the 17 genes are listed in Table S3.

### The *sdd3*∆/∆ mutant is defective in biofilm formation

**Fig 2:**
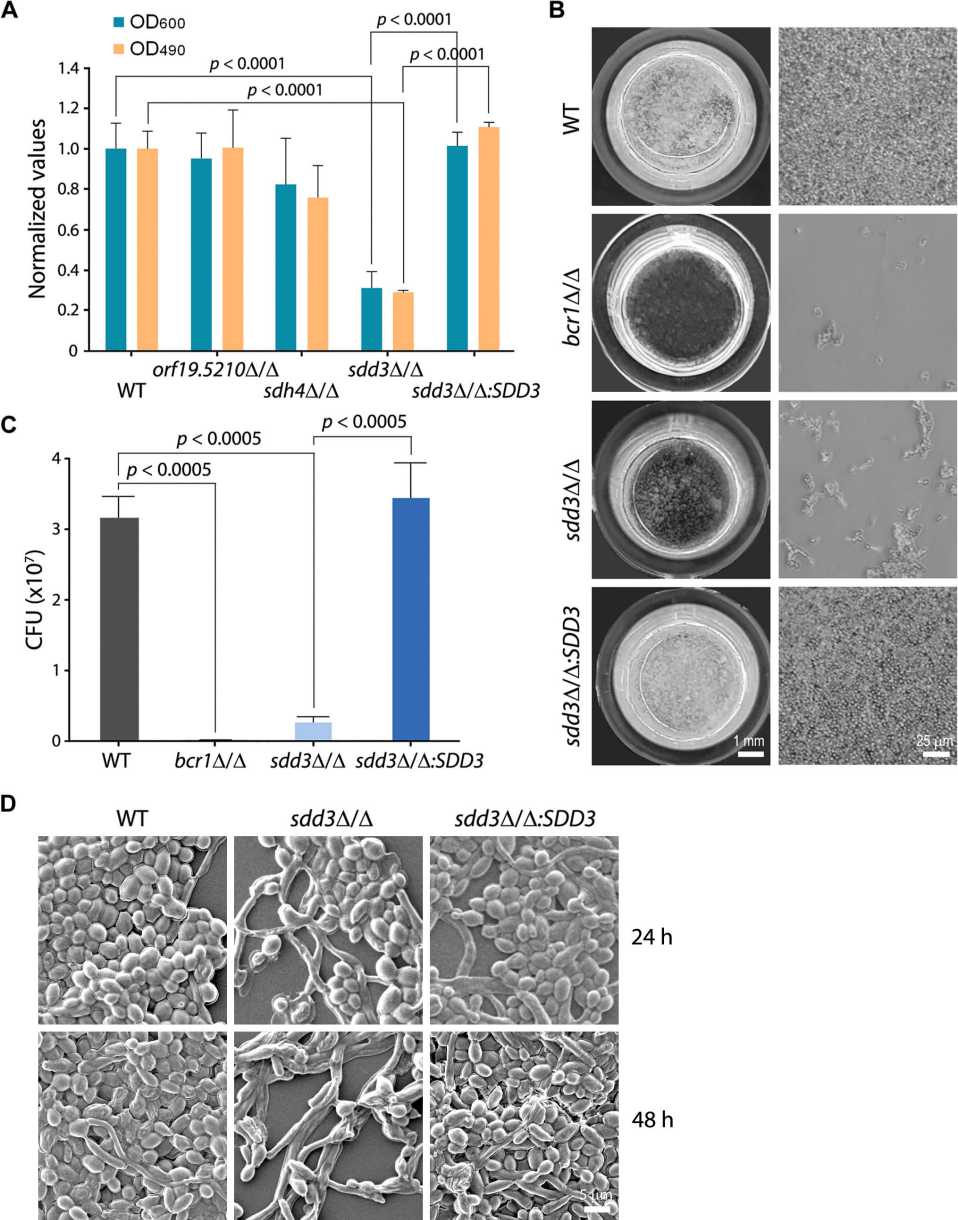
The *sdd3*∆/∆ mutant is defective in biofilm formation. (**A**) Assessment of biofilms formed by WT (BWP17UH), *orf19.5210*∆/∆ (GZY1389), *sdh4*∆/∆ (GZY1387), *sdd3*∆/∆ (GZY1399), and *sdd3*∆/∆*:SDD3* (GZY1412). The density of biofilms was measured at OD_600_, and the metabolic activity of biofilms was measured using the XTT assay at OD_490_. All values were normalized against the WT strain. (**B**) Microscopic examination of biofilms formed by WT, *bcr1*∆/∆ (GZY1094), *sdd3*∆/∆, and *sdd3*∆/∆*:SDD3* at 48 h. (**C**) Counting of viable cells in the biofilms formed by WT, *bcr1*∆/∆, *sdd3*∆/∆, and *sdd3*∆/∆*:SDD3*. Cells from each biofilm were dispersed, diluted, and spread onto yeast extract dextrose (YPD) plates to calculate colony-forming units (CFU). Data shown are the average of three independent experiments, and error bars represent standard error mean (SEM). (**D**) Scanning electron microscopy images of biofilms formed by WT, *sdd3*∆/∆, and *sdd3*∆/∆*:SDD3* after 24 and 48 h development.

To ascertain that the observed abnormal biofilm phenotypes are not unique to the haploid background, three genes, *ORF19.5210*, *SDH4*, and *SDD3* (*ORF19.6693*), were selected for further investigation. Both alleles of these genes were deleted using the *URA3* and *HIS1* selectable markers in the diploid strain BWP17, and the resulting mutants were subjected to biofilm assays. The biofilms formed by *orf19.5210*∆/∆, *sdh4*∆/∆, and *sdd3*∆/∆ mutants were assessed using OD_600_ measurement and the 2,3-bis-(2-methoxy-4-nitro-5-sulfophenyl)−2H-tetrazolium-5-carboxanilide, disodium salt (XTT) assay, which measures the metabolic activity of the biofilm. The results showed that the *sdd3*∆/∆ mutant exhibited similar biofilm defects to its corresponding haploid insertional mutant (Fig. 2A). Although *orf19.5210*∆/∆ and *sdh4*∆/∆ also exhibited reduced biofilm formation compared to WT, the differences were not significant (Fig. 2A). To further confirm that *SDD3* is indeed required for biofilm formation, a WT copy of *SDD3* was introduced into the *sdd3*∆/∆ mutant. The resulting strain (*sdd3*∆/∆:*SDD3*) regained the ability to form a normal biofilm like the WT strain (Fig. 2A), confirming that Sdd3 is required for proper biofilm formation.

Next, we further examined biofilms formed by the *sdd3*∆/∆ mutant through microscopic inspection (Fig. 2B) and colony-forming unit (CFU) counting (Fig. 2C). In contrast to the WT strain that produced a dense layer of cells at the bottom of the well, the *sdd3*∆/∆ mutant only formed loosely scattered cell clusters slightly denser than those formed by the *bcr1*∆/∆ mutant. Consistently, the CFU count from the *sdd3*∆/∆ biofilm was only about 10% of that from the WT biofilm. Again, introducing a copy of *SDD3* into the *sdd3*∆/∆ mutant restored a normal biofilm, with a CFU count comparable to the WT biofilm.

Finally, we used scanning electron microscopy (SEM) to examine the architecture of biofilm. After 24 h of development, the WT strain formed a biofilm primarily composed of densely packed yeast cells, with a small number of filaments. In contrast, the biofilm formed by *sdd3*∆/∆ was thin and loose, with significantly fewer cells and a higher percentage of filamentous cells compared to the WT biofilm. At 48 h, the WT biofilm was a mixture of tightly interwoven yeast and hyphal cells, while the *sdd3*∆/∆ biofilm remained as a loose network composed of some long filaments and scattered yeast cell clusters. The biofilm of the rescued *sdd3*∆/∆:*SDD3* strain exhibited an architecture similar to that of the WT strain (Fig. 2D). Taken together, these results demonstrate that Sdd3 plays an essential role in biofilm formation.

### The *sdd3*∆/∆ mutant has reduced chitin content

**Fig 3:**
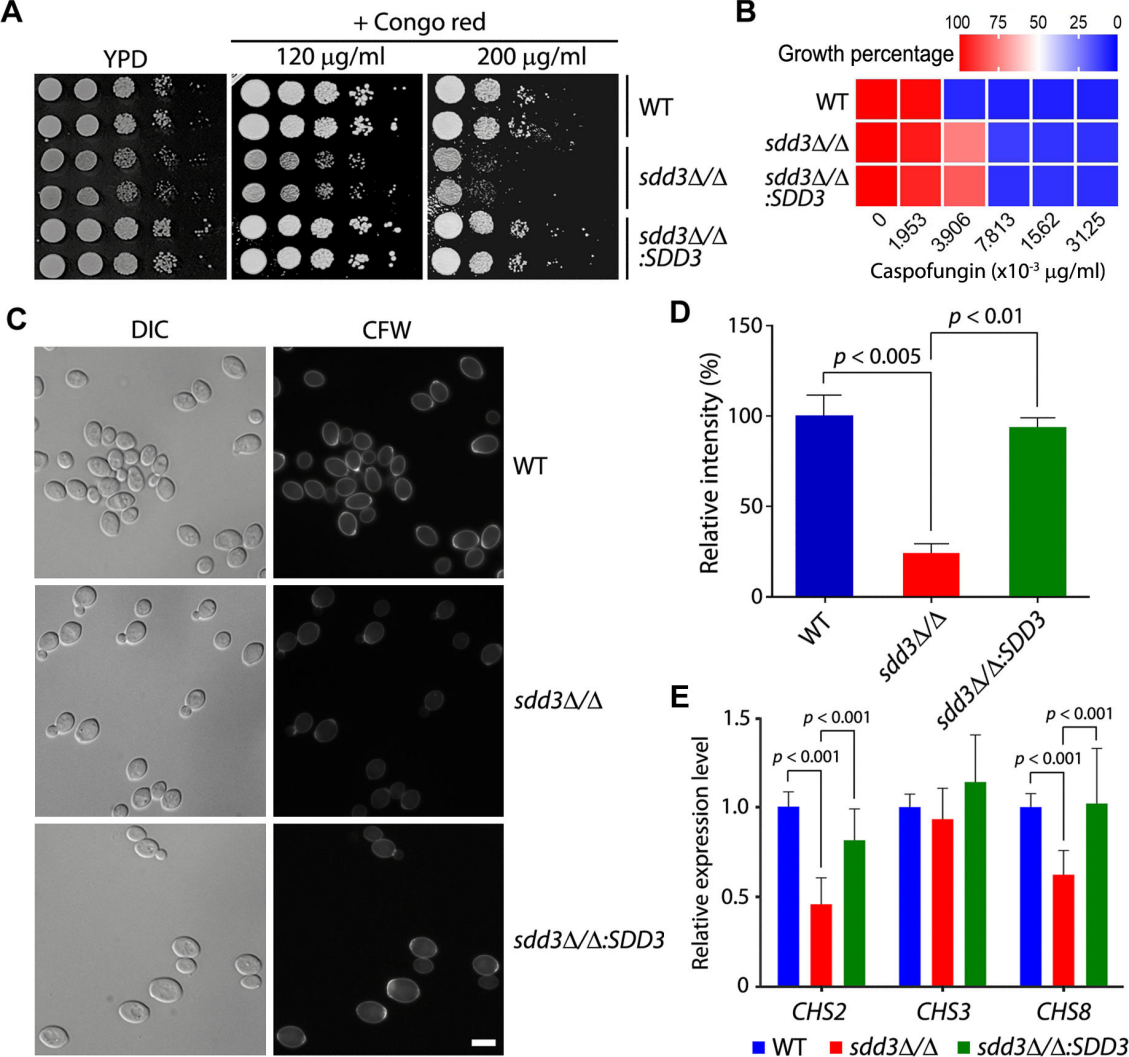
The *sdd3*∆/∆ mutant has reduced chitin content. (**A**) Sensitivity test of *sdd3*∆/∆ cells to CR. YPD cultures of WT, *sdd3*∆/∆, and *sdd3*∆/∆*:SDD3* were adjusted to OD_600_ = 1.0, serially diluted (1:10), and spotted onto YPD plates containing 0, 120, and 200 µg/mL of CR, respectively. The plates were incubated at 30°C for 2 days. (**B**) Sensitivity test of *sdd3*∆/∆ cells to caspofungin by MIC assay. Approximately 500 cells of WT, *sdd3∆/∆*, and *sdd3∆/∆* were added to 200 µL of YPD containing twofold serial dilutions of caspofungin and incubated at 30°C for 48 h. Growth was assessed by measuring OD_600_, and the values were normalized to the no-drug control for each strain. (**C**) Examination of chitin content by CFW staining. WT, *sdd3*∆/∆, and *sdd3*∆/∆*:SDD3* cells were cultured in glucose minimal medium (GMM), collected, and washed with PBS. The same number of cells (10^7^ cells/mL in PBS) were incubated with 50 µg/mL of CFW at room temperature (RT) for 2 h, followed by microscopic examination under fluorescence (CFW) and differential interference contrast (DIC) optics. Bar, 5 µm. (**D**) Quantitative analysis of fluorescence intensity in CFW-stained cells. The fluorescence intensities of ≥80 cells from each strain were measured using ImageJ, and the average intensity per cell in each group was calculated. The relative fluorescence intensities of *sdd3*∆/∆ and *sdd3*∆/∆ cells, compared to WT cells, are presented as percentages. The relative intensity values are the means of three independent measurements, and error bars represent standard error mean (SEM). (**E**) Examination of *CHS2*, *CHS3*, and *CHS8* expression levels by quantitative RT-PCR (qRT-PCR). Total RNA was extracted from yeast cultures of WT, *sdd3*∆/∆, and *sdd3*∆/∆*:SDD3* to perform qRT-PCR for *CHS2*, *CHS3*, and *CHS8. ACT1* was included in all qRT-PCR analyses for normalization. Data shown are the average of three independent experiments, and error bars represent SEM.

Biofilm formation is known to be highly dependent on cell surface properties, especially the cell wall (39). To determine whether the defective *sdd3*Δ/Δ biofilm results from altered cell wall properties, we assessed the susceptibility of *sdd3*Δ/Δ cells to Congo Red (CR), a cell wall-perturbing agent that interacts with chitin and β-glucans (40). Compared to the WT strain, the *sdd3*∆/∆ mutant was hypersensitive to CR, particularly at the higher concentration of 200 µg/mL (Fig. 3A). Furthermore, the rescued strain *sdd3*∆/∆:*SDD3* did not exhibit CR sensitivity, demonstrating that the CR sensitivity of the *sdd3*∆/∆ mutant is attributable to the loss of Sdd3. We found that the *sdd3*∆/∆ mutant exhibited similar susceptibility to the antifungal drug caspofungin as the WT strain, with no significant difference in their minimum inhibitory concentrations (MICs) (Fig. 3B). Caspofungin disrupts cell wall integrity by specifically inhibiting β-1,3-glucan synthase (41). Therefore, this result suggests that the deletion of *SDD3* has no significant effect on glucan synthesis. Next, we stained cells with the chitin-specific dye Calcofluor White (CFW) and examined them using a fluorescence microscope. Under the same staining and exposure conditions, *sdd3*∆/∆ cells exhibited much weaker fluorescent signals compared to the WT and *sdd3*∆/∆:*SDD3* cells (Fig. 3C). Quantitative analysis revealed that *sdd3*∆/∆ cells showed a 70%–76% reduction in fluorescence intensity compared to WT and *sdd3*∆/∆:*SDD3* cells (Fig. 3D), indicating a dramatic decrease in chitin content in *sdd3*∆/∆ cells.

To determine whether the reduced chitin content in the *sdd3*∆/∆ mutant results from decreased expression of *CHS2*, *CHS3*, and *CHS8*, the major chitin synthase genes in *C. albicans*, we quantified the expression of these genes using real-time quantitative RT-PCR (qRT-PCR). The results demonstrated that the expression of both *CHS2* and *CHS8*, but not *CHS3*, was significantly downregulated in the *sdd3*∆/∆ mutant (Fig. 3E).

Taken together, our results reveal that the deletion of *SDD3* downregulates the expression levels of *CHS2* and *CHS8*, resulting in reduced chitin content in the mutant cells.

### The activation of Rho1 and the PKC-MAPK pathway suppresses the chitin and biofilm defects of the *sdd3*∆/∆ mutant

**Fig 4:**
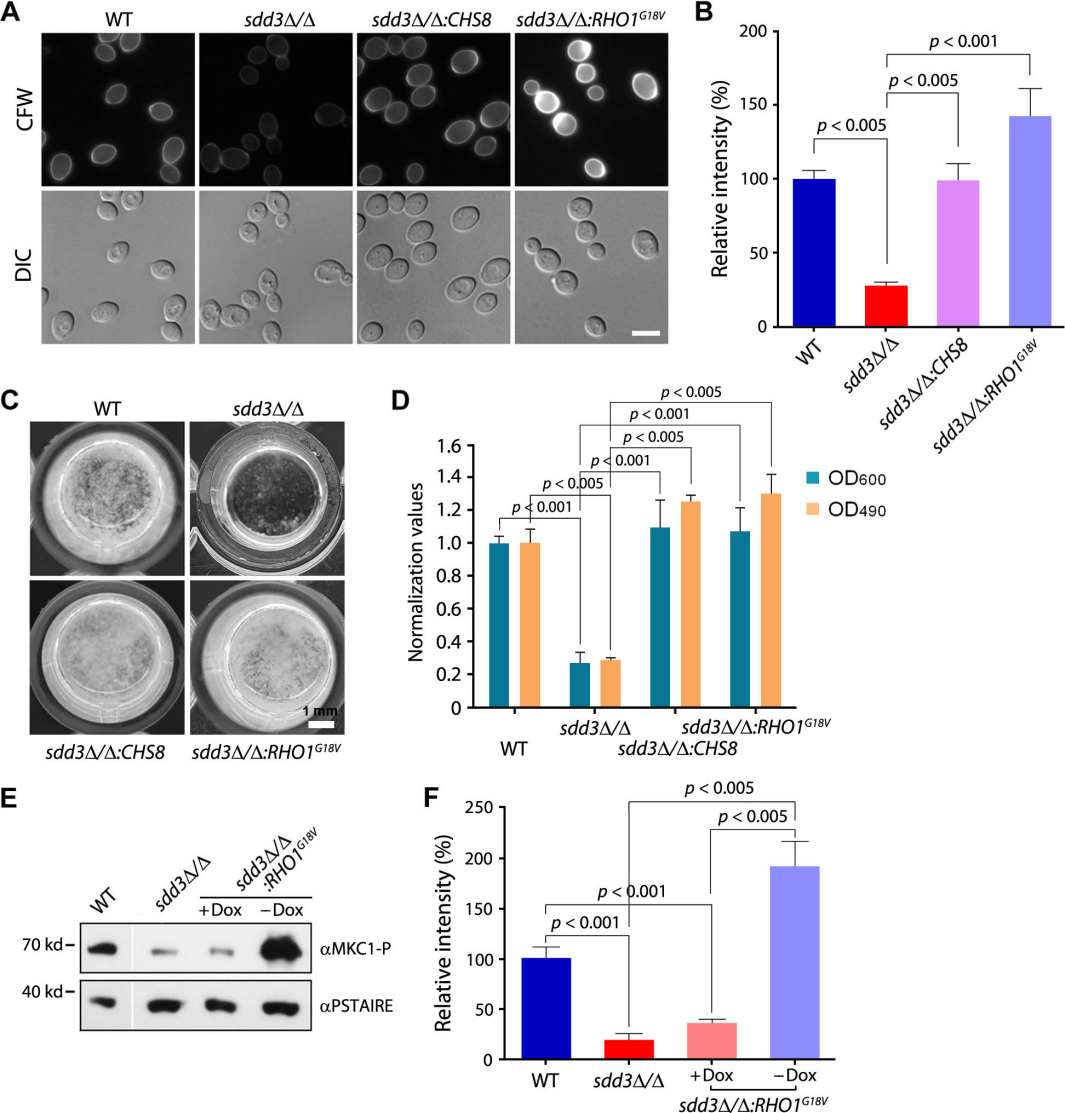
Activation of Rho1 and the PKC-MAPK pathway rescues the chitin and biofilm defects of the *sdd3*∆/∆ mutant. (**A**) Suppression of *sdd3*∆/∆ chitin defect by overexpressing *CHS8* or *RHO1^G18V^*. WT, *sdd3*∆/∆, *sdd3*∆/∆:*CHS8* (GZY1522), and *sdd3*∆/∆:*RHO1^G18V^* (GZY1457) cultures were adjusted to 10^7^ cells/mL in PBS. Cells were then incubated with 50 µg/mL of CFW at RT for 2 h, washed, and examined under a fluorescent microscope. Bar, 5 µm. (**B**) Quantitative analysis of fluorescence intensity of CFW-stained cells. The fluorescence intensities of cells (≥ 80 for each strain) were measured, and the average intensity per cell in each group was calculated. The relative fluorescence intensities of cells against that of WT cells are shown. The values are the means of three independent measurements, and error bars represent standard error mean (SEM). (**C**) Microscopic examination of biofilms formed by WT, *sdd3*∆/∆, *sdd3*∆/∆:*CHS8*, and *sdd3*∆/∆:*RHO1^G18V^*at 48 h. (**D**) Quantification of 72 h biofilms formed by WT, *sdd3*∆/∆, *sdd3*∆/∆:*CHS8*, and *sdd3*∆/∆:*RHO1^G18V^* by OD_600_ measurement and the XTT assay (OD_490_). All values were normalized against the WT strain. (**E**) Detection of Mkc1 phosphorylation levels in WT, *sdd3*∆/∆, and *sdd3*∆/∆:*RHO1^G18V^* (±50 µg/mL Dox) by western blotting (WB). Cells were harvested from glucose minimal medium (GMM) cultures, and protein lysates were prepared. Proteins were separated by SDS-PAGE and subjected to immunoblotting with αMKC1-P (detecting phosphorylated Mkc1) and αPSTAIRE (detecting the loading control Cdc28). Figures show the representative results of three repeated experiments. (**F**) Quantification of Mkc1 phosphorylation levels. The density of a protein band (measured by ImageJ) detected with αMKC1-P was normalized against the density of the same protein band detected with αPSTAIRE to represent the relative phosphorylation level. For comparison, the phosphorylation levels of Mkc1 in other conditions against that in WT were calculated and plotted. The data show the average of three independent experiments, and error bars represent SEM.

Since the *sdd3*∆/∆ mutant exhibits both defective biofilm formation and significantly reduced chitin levels, it is highly likely that the biofilm defects in *sdd3*∆/∆ result from the reduced chitin content. To test this possibility, we overexpressed either the major chitin synthase gene *CHS8* or the constitutively active form of *RHO1* (*RHO1^G18V^*) from the doxycycline (Dox)-repressible promoter *TetOff* (42) in the *sdd3*∆/∆ mutant to increase the chitin level. We then subjected these cells to biofilm formation assays. Rho1 belongs to the superfamily of small GTP-binding proteins that cycle between an active GTP-bound form and an inactive GDP-bound form to perform biological functions (43). It is a molecular switch that controls multiple downstream targets and acts via the PKC-MAPK pathway to activate chitin synthase expression (44). Our results demonstrated that *CHS8* overexpression restored the chitin content to the WT level in *sdd3*∆/∆ cells, while the overexpression of *RHO1^G18V^* in the *sdd3*∆/∆ mutant increased the chitin content to an even higher level (Fig. 4A and B). Moreover, the *sdd3*∆/∆ mutant overexpressing either *CHS8* or *RHO1^G18V^* produced normal biofilms indistinguishable from those of WT cells (Fig. 4C and D), indicating that the biofilm defects in the *sdd3*∆/∆ mutant are primarily attributable to the chitin deficiency. The suppression of the *sdd3*∆/∆ biofilm defect by *RHO1^G18V^* overexpression also suggests that Rho1 functions downstream of Sdd3 in the regulation of biofilm formation.

Along the PKC-MAPK pathway, the protein kinase C Pkc1 is the first molecule to be activated by Rho1 (44). Pkc1 activation triggers a cascade of MAP kinase phosphorylation, including Bck1 (MAPKKK), Mkk1 (MAPKK), and Mkc1 (MAPK) (29, 45). Phosphorylated Mkc1 then activates the transcription factor Rlm1 to control the expression of chitin synthase genes (46, 47). To investigate whether the reduced chitin content observed in the *sdd3*∆/∆ mutant is due to the downregulation of the PKC-MAPK pathway, we examined the Mkc1 phosphorylation level, which reflects the extent of activation of the MAPK pathway (48), in *sdd3*∆/∆ mutant cells. Compared to the WT strain, the *sdd3*∆/∆ mutant showed a markedly reduced phosphorylation level of Mkc1 (Fig. 4E and F). In contrast, the Mkc1 phosphorylation level in the *sdd3*∆/∆ mutant was dramatically increased when *RHO1^G18V^* was overexpressed (*sdd3*∆/∆:*RHO1^G18V^*, −Dox) but remained low when *RHO1^G18V^* expression was inhibited (*sdd3*∆/∆:*RHO1^G18V^*, +Dox).

Collectively, our data suggest that Sdd3 acts through Rho1 and the PKC-MAPK pathway to regulate chitin synthesis, thereby maintaining chitin content at a proper level essential for biofilm formation.

### Sdd3 interacts with Bem2 to regulate Rho1 activity

**Fig 5:**
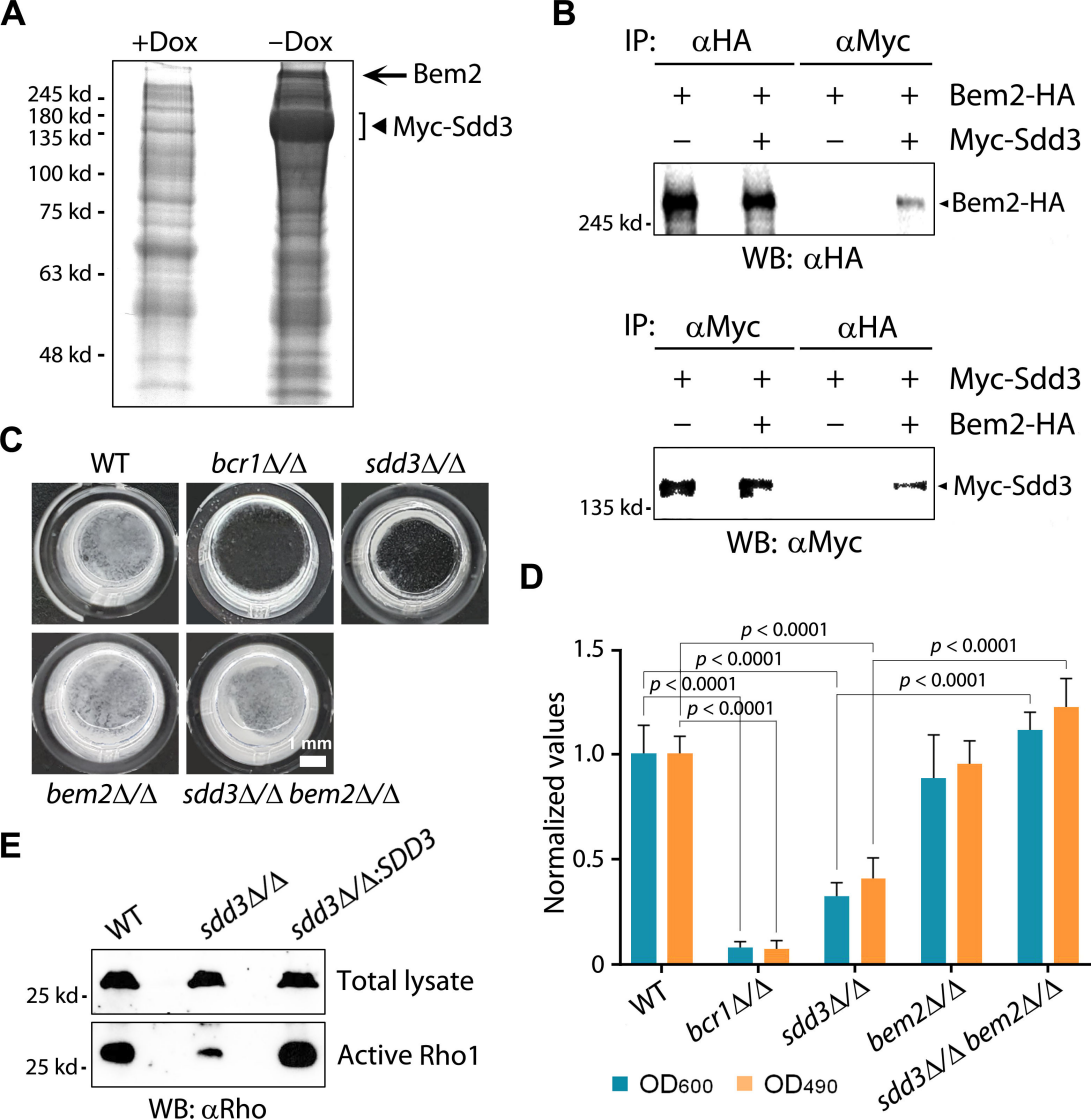
Sdd3 interacts with Bem2 to maintain the active form of Rho1. (**A**) Immunopurification of Myc-Sdd3-associated proteins. Protein extracts prepared from cells (GZY1467) expressing Myc-Sdd3 (−Dox) and cells without Myc-Sdd3 expression (+Dox) were incubated with αMyc, and the immunoprecipitated proteins were separated by SDS-PAGE followed by gel staining. A strong band uniquely presented in Myc-Sdd3 expressing cells (arrow) was excised for identification by mass spectrometry. (**B**) Validation of the physical interaction between Sdd3 and Bem2 by co-immunoprecipitation. Protein extracts prepared from cells expressing Myc-Sdd3 (GZY1467) or Bem2-HA (GZY1496) alone, or both (GZY1495), were subjected to immunoprecipitation with either αHA or αMyc and probed with αMyc and αHA. (**C**) Microscopic examination of biofilms formed by WT, *bcr1*∆/∆, *sdd3*∆/∆, *bem2*∆/∆, and *sdd3*∆/∆ *bem2*∆/∆ cells at 48 h. (**D**) Quantification of 72 h biofilms formed by WT, *bcr1*∆/∆, *sdd3*∆/∆, *bem2*∆/∆, and *sdd3*∆/∆ *bem2*∆/∆ using OD_600_ measurement and the XTT assay (OD_490_). All values were normalized against the WT strain. (**E**) Comparison of active Rho1 between WT and the *sdd3*∆/∆ mutant. An equal number of cells was harvested from glucose minimal medium (GMM) cultures of WT, *sdd3*∆/∆, and *sdd3*∆/∆*:SDD3* and split into two halves. Total protein lysates prepared from one half were directly subjected to western blotting (WB) with αRho to detect total Rho1. Protein extracts prepared from the other half were used to pull down active Rho1, followed by WB with αRho. Figures show the representative results of three repeated experiments.

To understand the molecular mechanism by which Sdd3 regulates Rho1 and the PKC-MAPK signaling pathway, we performed immunopurification to identify potential Sdd3-interacting proteins. Overexpressed Myc-Sdd3 was immunoprecipitated, and its associated proteins were eluted, separated by SDS-PAGE, and stained for visualization. In the control experiment, protein extract from cells without Myc-Sdd3 expression was used for immunoprecipitation. On the stained gel, a thick protein band with a molecular weight higher than 245 kd was detected in the Myc-Sdd3-expressing sample but not in the control sample, suggesting that it could be a Sdd3-specific binding protein (Fig. 5A). We excised this band for mass spectrometry (MS) analysis and identified the protein as Bem2.

To confirm that Bem2 indeed physically interacts with Sdd3, we tagged Bem2 with hemagglutinin (HA) and performed co-immunoprecipitation (co-IP) with Myc-Sdd3. When immunoprecipitated with the anti-Myc antibody (αMyc), Bem2-HA could be pulled down in the presence of Myc-Sdd3 but not in its absence. Conversely, when immunoprecipitated with αHA, Myc-Sdd3 could be pulled down only in the presence of Bem2-HA (Fig. 5B). Taken together, these results demonstrate a physical interaction between Sdd3 and Bem2.

Bem2 is the GAP of Rho1, converting the active Rho1-GTP to the inactive Rho1-GDP (49). As both Sdd3 and active Rho1 are required for biofilm formation and Rho1 functions downstream of Sdd3 (Fig. 2B and 4C), we hypothesize that Sdd3 binding may inhibit Bem2’s GAP activity to prevent the conversion of Rho1 from the active form to the inactive form. If this is the case, in the absence of Sdd3, Bem2 would become hyperactivated, resulting in the depletion of Rho1-GTP and downregulation of PKC-MAPK signaling, which ultimately leads to chitin deficiency and biofilm defects. Also, deleting *BEM2* should suppress the biofilm defects in the *sdd3*∆/∆ mutant. Indeed, we found that the *sdd3*∆/∆ *bem2*∆/∆ mutant regained the ability to form normal biofilms (Fig. 5C and D).

To further demonstrate that Sdd3 regulates Rho1 activity, we compared the amount of active Rho1 between the WT strain and the *sdd3*∆/∆ mutant. Equal amounts of cells were collected from each culture of the WT, *sdd3*∆/∆, and *sdd3*∆/∆*:SDD3* strains and split into two halves. Total protein lysates were prepared from one half using urea lysis buffer and subjected to SDS-PAGE and immunoblotting using the anti-Rho antibody (αRho), which detects total Rho1, including both active and inactive forms. Figure 5E shows that equal amounts of total Rho1 were detected in three samples. Next, protein lysates were prepared from the second half of the samples, and active Rho1 was pulled down for detection by using the Active Rho Pull-Down and Detection Kit. The results showed that the *sdd3*∆/∆ mutant had much less active Rho1 compared to the WT and *sdd3*∆/∆*:SDD3* strains (Fig. 5E), suggesting that most active Rho1 was converted to the inactive form by Bem2 due to the absence of Sdd3. Taken together, our results demonstrate that Sdd3 plays a critical role in maintaining the active form of Rho1 via its inhibitory interaction with Bem2.

### The *sdd3*∆/∆ mutant is virulent in the mouse model of systemic infection

**Fig 6:**
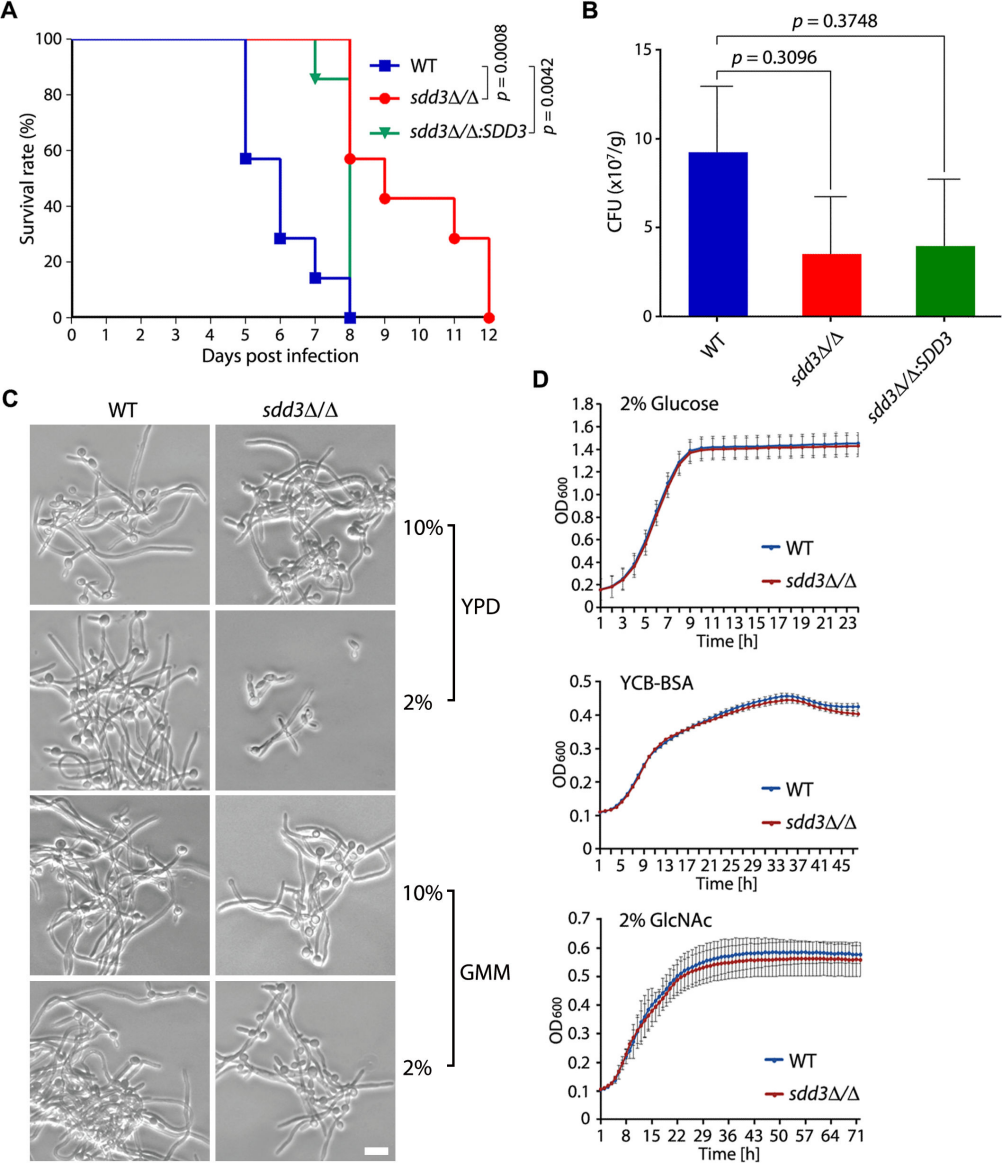
The *sdd3*∆/∆ mutant is virulent in the mouse model of systemic infection. (**A**) Virulence analysis of WT (BWP17UH), *sdd3*Δ/Δ, and *sdd3*Δ/Δ*:SDD3* strains. Each of the 8–10 weeks old BALB/c mice (*n* = 9 per group) was injected with 8 × 10^5^ yeast cells of a strain via the tail vein and monitored for survival till 15 days post-infection. (**B**) Fungal burden in the kidney of mice infected with WT, *sdd3*Δ/Δ, and *sdd3*Δ/Δ*:SDD3*. Two mice from each group were sacrificed 48 h post-infection. Kidneys were harvested, weighed, and homogenized for CFU enumeration. (**C**) Hyphal growth of WT and *sdd3*Δ/Δ cells. WT and *sdd3*Δ/Δ cells were cultured in YPD and glucose minimal medium (GMM) and induced for hyphal growth with 2% and 10% of fetal bovine serine (FBS) and incubation at 37°C for 2 h. Bar, 5 µm. (**D**) Growth curves of WT and *sdd3*Δ/Δ cultured in different media. WT and *sdd3*Δ/Δ were cultured in the indicated medium at 30°C overnight and diluted with the same medium to OD_600_ = 0.1. The diluted cultures were aliquoted to a 96-well microplate and incubated at 37°C. Growth was recorded by measuring OD_600_ in a microplate reader at 1 h intervals for up to 72 h. Graphs show the mean ± SD of three independent biological replicates.

Finally, we examined the virulence of the *sdd3*∆/∆ mutant. Approximately 8 × 10^5^ cells of WT, *sdd3*∆/∆, and *sdd3*∆/∆:*SDD3* strains were injected into 8–10 weeks old BALB/c mice (*n* = 9 per group) via the tail vein. Two mice from each group were sacrificed for kidney CFU counting 2 days post-infection, and the remainder was monitored for survival up to 12 days. Mice infected with the WT or the *sdd3*∆/∆:*SDD3* strain began to die on days 5 and 7, respectively, and all died within 4 and 2 days, respectively. In comparison, mice infected with the *sdd3*∆/∆ mutant began to die on day 8, and all died within 4 days (Fig. 6A). These results demonstrate that *sdd3*∆/∆ cells kill mice at a similar rate like WT cells but took a longer time (3 more days) to initiate the killing. Kidney CFU counting revealed fewer CFUs in *sdd3*∆/∆ and *sdd3*∆/∆:*SDD3*-infected mice than in WT-infected mice, but the differences were not significant (Fig. 6B).

Consistent with its virulence, the *sdd3*∆/∆ mutant was able to undergo hyphal growth like the WT strain upon serum induction in both YPD and glucose minimal medium (GMM), although the hyphal growth was slightly compromised in YPD with 2% serum (Fig. 6C). Furthermore, the *sdd3*∆/∆ mutant exhibited a growth curve similar to that of the WT strain, whether cultured in the rich medium YPD or in the physiologically relevant media yeast carbon base-bovine serum albumin (YCB-BSA) and GlcNAc (Fig. 6D).

## DISCUSSION

In this study, we report the identification of *ORF19.6693*, an uncharacterized *C. albicans* gene orthologous to the budding yeast *SDD3* gene, as a positive regulator of biofilm formation. We have further elucidated the molecular pathways by which Sdd3 regulates this process: Sdd3 binds to and inhibits Bem2, the GAP of the Rho1 GTPase, thereby sustaining the active form of Rho1 at a normal level. The active Rho1 then triggers its downstream PKC-MAPK pathway, which controls the transcription of chitin synthesis genes. This regulation ensures that chitin content remains at an appropriate level, a critical factor for biofilm formation.

*SDD3* was identified during a screen of a haploid transposon-insertion library for mutants defective in biofilm formation. Since its discovery about 10 years ago, haploid *C. albicans* has become a powerful genetic tool for conducting genome-scale studies of this pathogen (50–54). Many new genes that play a critical role in diverse traits important for the virulence and pathogenicity of *C. albicans* have been identified, including those involved in biofilm formation (55), the white-opaque switch (42), azole resistance (51), amphotericin B resistance (56), the mechanism of action of antifungal agents (57), and antifungal susceptibility (58). Additionally, novel tools and platforms for genetic interaction analysis have also been established (52–54). More recently, screening of the haploid mutant library identified cytochrome *c* (Cyc1) as a novel factor governing the yeast-hyphal transition and regulating metabolic adaptation (59). We anticipate that future innovative applications of haploid *C. albicans* will lead to even more discoveries.

The initial screen identified 5 biofilm-enhancing and 12 biofilm-repressing haploid mutants. Likely due to differences in the genetic background between haploid and diploid *C. albicans* strains (50), as well as differences in the effects of transposon insertion vs gene deletion mutations, biofilm defects observed in some haploid mutants could not be verified in the diploid background, such as the *sdh4* and *orf19.5210* mutants. However, the biofilm defect of the haploid *sdd3* mutant was reproduced in the diploid *sdd3*Δ/Δ strain. Interestingly, deletion of *SDD3* has been reported to eliminate biofilm formation in *Saccharomyces cerevisiae* as well, although the underlying molecular mechanism was unclear (60). The apparent evolutionary conservation of *SDD3*’s role in biofilm formation prompted us to conduct a comprehensive investigation of the function of Sdd3 in *C. albicans* biofilm formation. Our results strongly support the essential role of Sdd3 in biofilm formation in *C. albicans*. Both the haploid transposon-insertion *sdd3* mutant and the diploid *sdd3*Δ/Δ mutant formed defective biofilms on the surface of polystyrene plates. While the WT strain formed biofilms consisting of densely packed cells, primarily yeast cells with some filamentous cells, the *SDD3* mutant grew on the surface as loosely scattered yeast cell clusters connected by filaments. This indicates that the deletion of *SDD3* significantly disrupts the development of biofilms. Importantly, all the defects observed in *sdd3*Δ/Δ biofilms were rectified by reintroducing a WT copy of *SDD3*.

It remains unclear which stage of biofilm development is impaired in the *sdd3*Δ/Δ mutant. We observed that the initial adhesion of *sdd3*Δ/Δ yeast cells to the surface appears normal, and the mutant cells can also switch to hyphal growth similarly to WT cells. Differences start to manifest after 24 h when cells start to lose contact with the surface or with each other, resulting in many cells floating in the medium. We suspect that the severe chitin deficiency of the *sdd3*Δ/Δ mutant may cause significant changes in cell surface properties, which weakens cells’ adhesion to substrates and intercellular interactions during the maturation stage of biofilm formation. Additionally, the chitin synthesis defect may activate compensatory or salvage mechanisms (61), resulting in elevated synthesis of other cell wall components, as well as components of the ECM, including approximately 55% glycoproteins and 25% carbohydrates (largely α-mannan and β-1,6-glucan polysaccharides and, to a lesser extent, β-1,3-glucan) (35). These changes may affect the ability of the ECM to hold the cells together, leading to compromised biofilm structures. In agreement with our speculation, *sdd3*∆/∆ mutant cells exhibited an increased level of mannoproteins (Fig. S1), with the glucan contents likely remained unchanged (Fig. 3B). Unfortunately, our SEM analysis did not detect the ECM because the method we used does not preserve it due to the dehydration step of sample preparation (62). Further investigation is needed to fully understand the cell surface properties underlying the biofilm defects observed in the *sdd3*∆/∆ mutant.

The *sdd3*Δ/Δ mutant exhibits markedly reduced chitin content and defective biofilm formation. However, increasing the chitin level in the mutant by overexpressing chitin synthases restores normal biofilm formation. These observations support the essential role of chitin in biofilm formation. This is not surprising, as chitin is a crucial component of the cell wall. Traditionally, the importance of the cell wall in biofilm formation has primarily been attributed to cell wall-associated proteins (31–34), rather than to the structural components like chitin or glucan. The importance of chitin in biofilm formation was also implicated in earlier studies, which demonstrated that the *mkc1*Δ*/*Δ mutant of *C. albicans* formed biofilms with abnormal structures and fewer hyphal cells (25), and Mkc1 is the key kinase of the cell-wall integrity pathway that regulates the expression of chitin synthases (46, 47). Although the exact mechanisms by which chitin determines biofilm formation remain unknown, it is reasonable to believe that defects in chitin synthesis cause changes in cell wall components, architecture, and associated proteins, ultimately impacting biofilm formation.

**Fig 7:**
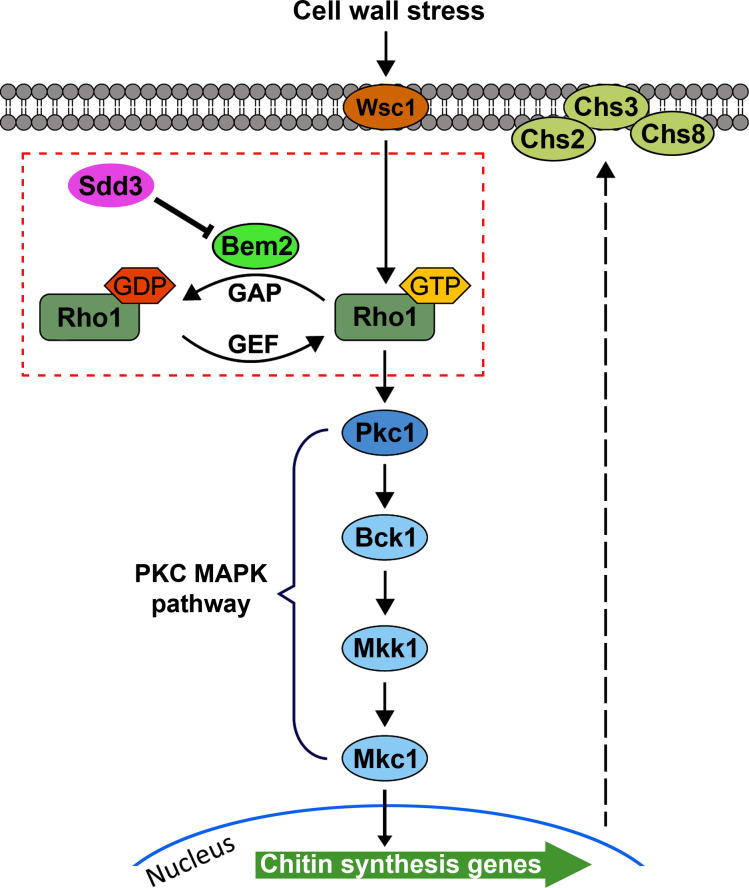
A model depicting the molecular mechanism by which Sdd3 regulates chitin synthesis and biofilm formation in *C. albicans*. Sdd3 binds to Bem2 and inhibits its GAP activity, thereby maintaining Rho1 in active, GTP-bound form. Active Rho1 signals through its downstream target, Pkc1, to activate the MAPK pathway, upregulating the transcription of chitin synthases. This leads to the production of sufficient chitin, which is essential for cell wall integrity and biofilm formation.

A key finding of this study is the elucidation of the entire signaling pathway linking Sdd3 to chitin synthesis. We discovered that Sdd3 regulates the activity of the Rho1 GTPase by inhibiting Bem2, the GAP of Rho1 GTPase that converts active Rho1 to its inactive form. Rho1 has multiple downstream effectors, one of which is Pkc1, which acts through the Mkc1 MAPK pathway to control the expression of chitin synthases. Therefore, we propose the following model: the inhibitory interaction of Sdd3 with Bem2 increases the amount of GTP-bound Rho1, which activates the PKC-MAPK pathway and stimulates the transcription of chitin synthases to maintain chitin production (Fig. 7). In support of this model, our results demonstrated that *sdd3*Δ/Δ cells had lower levels of GTP-Rho1 and phosphorylated Mkc1, along with downregulated chitin synthase genes and reduced chitin content. However, how Sdd3 inhibits the GAP activity of Bem2 remains unknown. It is possible that Sdd3’s binding to Bem2 triggers Bem2 degradation, alters it to an inactive conformation, or prevents Bem2 from binding to Rho1. Further investigations are needed to answer these questions.

In comparison to the extensive knowledge of the transcriptional networks that regulate biofilm formation, little is known about the signaling pathways that control it. This study not only identified a new biofilm regulator, Sdd3, in *C. albicans* but also established the Rho1-PKC-MAPK pathway through which Sdd3 governs biofilm formation by modulating chitin synthesis. Further exploration of other biofilm-related cell signaling pathways will enhance our understanding of this key virulence trait of *C. albicans*.

Biofilm formation by *C. albicans* is a complex process that can lead to life-threatening infections due to its intrinsic resistance and tolerance to standard antifungal drugs and the host immune system. Current antifungal therapies have minimal effects on biofilm formation, and there is no effective solution to this problem. Therefore, a deeper mechanistic understanding of biofilm formation and its regulation is important for developing antifungal drugs effective against biofilms. Targeting the biofilm-related pathways could serve as a promising strategy in combating *C. albicans* biofilm formation. Sdd3’s role in regulating the Rho1-PKC MAPK cell wall integrity pathway and biofilm formation suggests that it could be a potential target for anti-biofilm drugs. Furthermore, a BLAST search revealed that Sdd3 has no homolog in mammals.

## MATERIALS AND METHODS

### Strains, media, and growth conditions

Yeast strains and plasmids used in this study are described in Tables S1 and S2, respectively. Recombinant DNA manipulation was performed according to standard protocol. *Escherichia coli* DH10B (Invitrogen, Cat. 18290015) was used as the host strain for recombinant plasmids and cultured in Luria-Bertani (LB) broth (0.5% yeast extract, 1% tryptone, and 0.5% NaCl, pH 7.0) supplemented with 100 µg/mL ampicillin. Transformation of *C. albicans* was performed using Fast Yeast Transformation Kit (G-Biosciences, Cat. GZ-1) according to the manufacturer’s protocol. Gene deletion was verified by colony PCR as described previously (52). Yeast cells were routinely grown at 30°C in YPD (1% yeast extract, 2% peptone, and 2% glucose) or GMM (6.79 g/L yeast nitrogen base without amino acids, and 2% glucose) supplemented with appropriate amino acids and compounds (80 µg/mL uridine, 40 µg/ml arginine, 40 µg/mL histidine, 1 mg/mL 5-fluoroorotic acid, and 200 µg/mL nourseothricin) when necessary. Solid medium plates were prepared by adding 2% agar. For hyphal induction, overnight yeast culture was diluted 1:20 into fresh medium containing 2%–10% fetal bovine serum (Hyclone) and incubated at 37°C for durations as required.

### Transposon-mediated haploid mutant collection

Transposon-mediated insertional haploid mutants were generated according to procedures as described previously (36, 37). Briefly, the parental strain YW02 was inoculated into 5 mL of YPD medium containing 50 µg/mL doxycycline and incubated at 30°C overnight to induce the expression of *piggyBac* transposase. The cells were washed with fresh GMM medium and further cultured in 20 mL of GMM overnight to enrich the insertional haploid mutants. Finally, cells were diluted, spread onto GMM plates, and incubated at 30°C for 3 days to obtain independent single colonies.

### Biofilm formation and assessment

Haploid biofilm screening assay was performed as described previously with slight modifications (63). Single colonies of the transposon-insertional mutants were inoculated into 96-deep well plate (round bottom 2.2 mL polypropylene, Axygen) containing 400 µL of GMM. The plate was sealed with Breathe-Easy sealing membrane (Axygen) and incubated at 30°C for 22 h with shaking (180 rpm). The overnight cultures were diluted with fresh GMM to OD_600_ = 0.11–0.12 (measured using Tecan Infinite M200 Pro microplate reader). One hundred microliters of each cell suspension was then transferred to a flat-bottomed 96-well polystyrene plate (tissue culture-treated, Falcon). The plate was sealed with a sealing membrane and incubated for 1.5 h at 37°C with shaking at 75 rpm to allow cell adherence. Next, the non-adherent cells were removed, and each well was washed twice with 150 µL of PBS. Two hundred microliters of fresh GMM was added to each well, and the plate was incubated for 72 h at 37°C with shaking at 75 rpm. The medium was changed every 24 h to remove planktonic cells. After 72 h incubation, planktonic cells were aspirated from the wells, and the biofilms were washed twice with PBS. Biofilm formation assay for diploid strains was performed as described previously with slight modifications (55). Single colonies were inoculated in 2 mL of GMM and incubated overnight at 30°C with shaking at 180 rpm. Cells were washed twice with PBS, and the cell concentration was adjusted to 10^7^ cells/mL (in PBS) using a hemocytometer. Subsequent steps were performed as described in the haploid biofilm screening assay.

To visually assess biofilms, the 96-well polystyrene plate was mounted on the stage of an inverted microscope (Olympus IX70). High-magnification images of *C. albicans* cells were taken with a Motic Cam 10 MP camera and Motic Images Plus 2.0 software. To quantify biofilm density, the plate was read at the optical density of 600 nm using the microplate reader. Five independent locations per well were read to gain an average density. For counting of CFU, cells were scratched from each well, serially diluted, spread onto YPD plates, and incubated at 30°C for 2 days. The metabolic activity of biofilms was measured using XTT colorimetric assay (Biotium) according to the manufacturer’s protocol. One hundred fifty microliters of PBS and 25 µL of activated XTT solution (activation reagent:XTT solution = 1 : 200) were added to each well. The plate was then incubated at 37°C for 2 h with shaking in the dark. One hundred microliters of the supernatant was transferred to a new well, and the absorbance was read at 490 nm using the microplate reader. Five independent locations per well were read to gain an average density. All experiments were repeated independently at least three times.

### Scanning electron microscopy

The biofilm was washed twice with PBS and fixed with formalin solution (10% neutral buffered) for 2 h. After washing twice with deionized water, dehydration of the biofilm was performed using a series of ethanol/water mixtures (25%, 50%, 70%, 90%, and 100%). The dehydrated samples were then dried at room temperature (RT) overnight. SEM images were recorded using a JEOL JSM-7900F Schottky Field Emission Scanning Electron Microscope at an acceleration voltage of 5 kV. Samples were sputter coated with gold using a Cressington 208 HR High-Resolution Sputter Coater for improved conductivity.

### Congo Red sensitivity, chitin and mannoprotein staining, and fluorescence intensity quantification

For Congo Red sensitivity test, *C. albicans* strains were cultured in 2 mL of YPD at 30°C overnight. The cells were washed twice with PBS and adjusted to OD_600_ = 1.0. Cell suspensions were then diluted 10 times in series from 10^−1^ to 10^−5^. Three microliters of each diluted culture was then spotted onto YPD plates without or with the addition of Congo Red (120 and 200 µg/mL). The plates were incubated at 30°C for 24 h before visualization.

To stain chitin with CFW, *C. albicans* strains were cultured in 2 mL of YPD at 30°C overnight. Cells were washed twice with PBS and adjusted to 10^7^ cells/mL (in PBS) using a hemocytometer. The cells were then incubated with 50 µg/mL of CFW (Sigma-Aldrich Cat. 910090) in dark at RT for 2 h with shaking. The stained cells were washed twice with PBS followed by microscopic visualization. Samples were examined by Leica DM RXA2 fluorescence microscope equipped with a CoolSnap HQ2 digital camera (Roper Scientific). Images were acquired using the MetaMorph 7.5 software.

To stain mannoproteins with ConA-FITC, *C. albicans* strains were cultured in 2 mL of GMM at 30°C overnight, reinoculated into fresh GMM, and continued the growth till log phase. Cells were collected and washed three times with sterile PBS. The cells were then incubated with 100 µg/mL ConA-FITC (Sigma-Aldrich, C7642) for 45 min in the dark. After the staining, cells were washed three times in PBS. Finally, the cells were fixed in 0.3% sodium azide for 1 h and observed under the microscope.

Quantification of fluorescence intensity was carried out as described previously (46). Briefly, the acquired images were analyzed using the Image J software (https://imagej.nih.gov/ij/), and the relative intensity of a whole image was measured. Total cell numbers of the image were also counted and used to calculate the average intensity of a single cell. At least 80 individual cells from *>*3 images were measured and counted for each sample.

### RNA preparation and qRT-PCR

For preparation of total RNA, strains were cultured in 5 mL of YPD at 30°C overnight. Total RNA was extracted using hot acidic phenol method with slight modification to previous protocol (64). Briefly, the cell pellets were obtained by centrifugation in 1.7 mL tubes. Four hundred fifty microliters of acetate-ethylenediamine tetraacetic acid (AE) solution (50 mM sodium acetate, 10 mM EDTA, pH 5.2), 20 µL of 25% SDS, and 300 µL of acid phenol (pH 4.3) was added to the cell pellets and incubated at 65°C for 10 min with occasional vortex. Next, the tubes were placed on ice for 5 min and then centrifuged at 13,000 rpm at 4°C for 15 min. The aqueous layer was transferred to a clean 1.7 mL tube, and 500 µL of chloroform was added and vortexed vigorously. After centrifugation at 8,000 rpm for 10 min at 4°C, the aqueous layer was transferred to a clean 1.7 mL tube. An equal volume of 100% isopropanol and 1/10 vol of 3 M sodium acetate were added with gentle mixing by inversion. The mixture was then centrifuged at 13,000 rpm for 20 min at 4°C. The RNA pellets were washed twice with 70% ethanol and air dried. To remove any contaminated DNA, the RNA was treated with DNase I (New England Biolabs) for 30 min according to the manufacturer’s protocol. Finally, the total RNA was purified with Monarch RNA Cleanup Kit (New England Biolabs).

To perform qPCR, total RNA was reverse transcribed to cDNA using the LunaScript RT SuperMix Kit (New England Biolabs). qPCR was performed on a QuantStudio 5 thermal cycler (Thermo Fisher) using Luna Universal qPCR Master Mix (New England Biolabs). In addition to target genes, the expression level of *ACT1* was also measured for normalization. All experiments were repeated independently at least three times (three biological duplicates). Sequences of all qPCR primers are listed in Table S4.

### Protein extraction, western blotting, and co-IP

To extract proteins for direct western blotting (WB), strains were cultured in 5 mL GMM at 30°C for 1–2 days. Cells were collected into 2 mL screw-cap microcentrifuge tubes by brief centrifugation. Each cell pellet was resuspended into an equal volume of urea lysis buffer (9 M urea, 20 mM HEPES [pH 8.0], 1 mM sodium orthovanadate, 2.5 mM sodium pyrophosphate, and 1 mM beta-glycerophosphate) and an equal volume of acid-washed glass beads (Sigma-Aldrich). Cells were lysed by FastPrep-24 5G homogenizer (MP Biomedicals) for five cycles of 1 min at a speed of 6.0 m/s with 1.5 min pause. The lysed cells were then centrifuged at 14,000 rpm for 15 min at 4°C, and the supernatants were collected. Each protein lysate was mixed with 2× protein loading buffer (125 mM Tris-HCl [pH 6.8], 4% SDS, 20% glycerol, 200 mM dithiothreitol, and 0.02% bromophenol blue) and heated at 100°C for 8 min before being subjected to SDS-PAGE.

To perform IP, strains expressing Myc-Sdd3, Bem2-HA, or both were inoculated into 50 mL of YPD and incubated at 30°C overnight. Cells were collected, and yeast lysis buffer (50 mM Tris-HCl [pH 7.4], 150 mM KCl, and 1% NP-40) containing protease inhibitor cocktail (Roche) instead of urea lysis buffer was used for protein extraction as described above. The cell lysates were incubated with Myc-Trap Agarose beads (ChromoTek) or Anti-HA Affinity Gel (Sigma Aldrich) at 4°C for 2 h. The beads were then washed four times with ice-cold yeast lysis buffer, mixed with 1× protein loading buffer, and heated at 100°C for 8 min before being subjected to SDS-PAGE.

For WB, protein samples were separated by SDS-PAGE and transferred to a polyvinylidene difluoride (PVDF) membrane (Bio-Rad). The membrane was incubated with 5% milk-blocking solution (dissolved in PBS containing 0.1% Tween-20, PBST) at RT for 1 h or at 4°C overnight. After a brief rinse with PBST, the membrane was incubated in PBST containing a diluted primary antibody (monoclonal Myc and HA antibodies from Roche) at RT for 1 h, followed by three rounds of 5 min wash with PBST. The membrane was then incubated with PBST containing a diluted secondary antibody (horseradish peroxidase [HRP]-linked anti-mouse IgG, Amersham). After three rounds of 5 min wash with PBST, the membrane was immersed in West Pico Chemiluminescent Substrate (Thermo Scientific), and signals were detected with ChemiDoc Touch Imaging System (Bio-Rad). To re-probe the membrane with another primary antibody, all the bound antibodies were removed by Restore Western Blot Stripping Buffer (Thermo Scientific).

### Pulldown of Sdd3-interacting proteins and MS

To identify Sdd3-interacting proteins, the strain carrying *TetOff-Myc-SDD3* was inoculated into either YPD medium or YPD containing 50 µg/mL of doxycycline and incubated at 30°C overnight. Cells were then collected into multiple 2 mL screw-cap microcentrifuge tubes. Protein extracts were prepared with yeast lysis buffer (containing protease inhibitor cocktail) from both samples. The lysates from the same sample were pooled, mixed with 50 µL slurry of Myc-Trap Agarose beads, and incubated at 4°C for 3 h. The beads were then washed three times with ice-cold lysis buffer and finally resuspended into 40 µL of 1× protein loading buffer. Bead-associated proteins were eluted by heating at 100°C for 10 min, separated by SDS-PAGE, and stained using the One-Step Blue (Biotium).

For MS analysis, protein bands unique to the strain expressing Myc-Sdd3 were excised and washed, followed by reduction and alkylation of disulfide bonds prior to in-gel digestion with trypsin (65). Digested samples were analyzed by liquid chromatography-tandem mass spectrometry on a nanoACQUITY UPLC system (Waters) coupled to an Linear ion Trap Quadrupole (LTQ) Orbitrap Elite MS (Thermo Scientific) operated in data-dependent acquisition mode, with selection of the top 15 most intense precursors fragmented by collision-induced dissociation per scan. Spectra data were searched against *C. albicans* UniProtKB reference proteome using Proteome Discoverer 2.4, with Percolator node and target false discovery rate at *q*-value of 0.01 for protein identification.

### Detection of phosphorylated Mkc1 and active Rho1

To determine the phosphorylation levels of Mkc1 in WT, *sdd3*∆/∆, and *sdd3*∆/∆*:RHO1^G12V^*, these strains were cultured in GMM medium at 30°C overnight. Cells from each strains were collected, and protein extracts were prepared using urea lysis buffer. Equal amounts of protein extracts were separated by SDS-PAGE and subjected to WB with rabbit polyclonal PhosphoPlus p44/42 MAPK antibody (Cell Signaling) and reprobed with rabbit polyclonal PSTAIRE (Santa Cruz). An HRP-linked anti-rabbit IgG antibody (Amersham) was used as the secondary antibody for both primary antibodies.

To compare the amounts of active Rho1 in WT, *sdd3*∆/∆, and *sdd3*∆/∆*:SDD3*, these strains were cultured in GMM medium at 30°C overnight. Same amounts of cells from each culture were collected and split into two halves. Protein lysates were prepared from one-half samples using urea lysis buffer. At the same time, the other half of the samples were used to prepare protein lysates for the pulldown of active Rho1 using the Active Rho Pull-Down and Detection Kit (Thermo Scientific). Both total protein lysates from the first half and active Rho1 from the second half were separated by SDS-PAGE and transferred to a PVDF membrane, followed by immunoblotting with anti-Rho (Thermo Scientific) and HRP-linked anti-rabbit IgG as the primary and secondary bodies, respectively.

### Virulence and fungal burden analysis

Female BALB/c mice between 8 and 10 weeks old were used for virulence analysis. *C. albicans* strains were cultured overnight in YPD at 30°C and harvested by centrifugation. Cells were washed twice with PBS and adjusted to 4 × 10^6^ cells/mL using a hemocytometer. Two hundred microliters of the cell suspension was injected to each mouse via the tail vein. Infected mice (*n* = 9 per group) were monitored for survival up to 12 days. GraphPad Prism 9 was used to analyze survival, and the results were shown as Kaplan-Meyer curves. Survival curves were compared using the log-rank (Mantel-Cox) test, and *P*-values are shown in the graph. For CFU analysis, two mice of each group were euthanized at 48 h post-infection to harvest kidneys. The harvested kidneys were homogenized in PBS, serially diluted, and plated on YPD plates containing 100 µg/mL of ampicillin. The plates were incubated at 30°C for 24 to 48 h, and colony numbers were counted and expressed as CFU per gram.

### Growth curve measurement

To determine the growth of *C. albicans* strains in different media, the strains were cultured at 30°C overnight and then diluted to an OD_600_ of 0.1 in respective media. Each diluted culture was then aliquoted to three wells (200 µL/well) of a 96-well plate. Growth was recorded by measuring OD_600_ in a microplate reader (Infinite M200 Pro, TECAN) at 37°C. Measurements were performed at 1 h intervals for not more than 72 h. Each measurement was performed with three independent biological replicates, and the results were shown as the mean ± SD. The media used in the experiments were 2% glucose (YPD), 2% GlcNAc (6.79 g/L yeast nitrogen base without amino acids and 2% N-acetyl-glucosamine), and YCB-BSA (1.17% YCB, 0.5% BSA, and 2% glucose [pH 4.0]).

### MIC assay for caspofungin

Caspofungin was purchased from LKT laboratories (Cat no. C0274) and prepared as stock solutions according to the manufacturer’s instructions. MIC assay was performed using the broth microdilution method described in the Clinical and Laboratory Standards Institute (CLSI) document M38 in a 96-well plate format. A standardized amount of ~500 cells was inoculated into 200 µL of YPD containing twofold serially diluted caspofungin (1–0.001953 µg/mL) in a 96-well plate, and the plate was incubated at 30°C for 48 h. The optical density of cells per well was measured at OD_600_ using a microplate reader (Tecan Infinite M200 Pro). The relative growth ratios were calculated by normalizing OD_600_ values against the no-drug controls. The results were plotted as a heatmap using GraphPad Prism 9.

### Statistical analysis

Graph Pad Prism Software Version 6.00 was used for all statistical analyses. Probability values of *P* < 0.05 were considered statistically significant. Data were expressed as ±SD, unless otherwise stated. Results of biofilm development assay were analyzed by two-tailed unpaired *t* test. All experiments were repeated independently at least three times.

## Data Availability

All data needed to evaluate the conclusions in the paper are present in the paper and/or the supplemental materials.

## SUPPLEMENTAL MATERIAL

Supplemental material mbio.03283-24-s0001.docx Fig. 1 and Tables S1-S4.

ASM does not own the copyrights to Supplemental Material that may be linked to, or accessed through, an article. The authors have granted ASM a non-exclusive, world-wide license to publish the Supplemental Material files. Please contact the corresponding author directly for reuse.
