# Supplementary material for: Sdd3 regulates the biofilm formation of Candida albicans via the Rho1-PKC-MAPK pathway
Source: mBio. 2024 Dec 17;16(2):e03283-24. doi: 10.1128/mbio.03283-24 (PMC11796410; doi:10.1128/mbio.03283-24)
Supplement: Supplemental material — Fig. 1 and Tables S1-S4. [file mbio.03283-24-s0001.docx]

**Fig. S1**

**
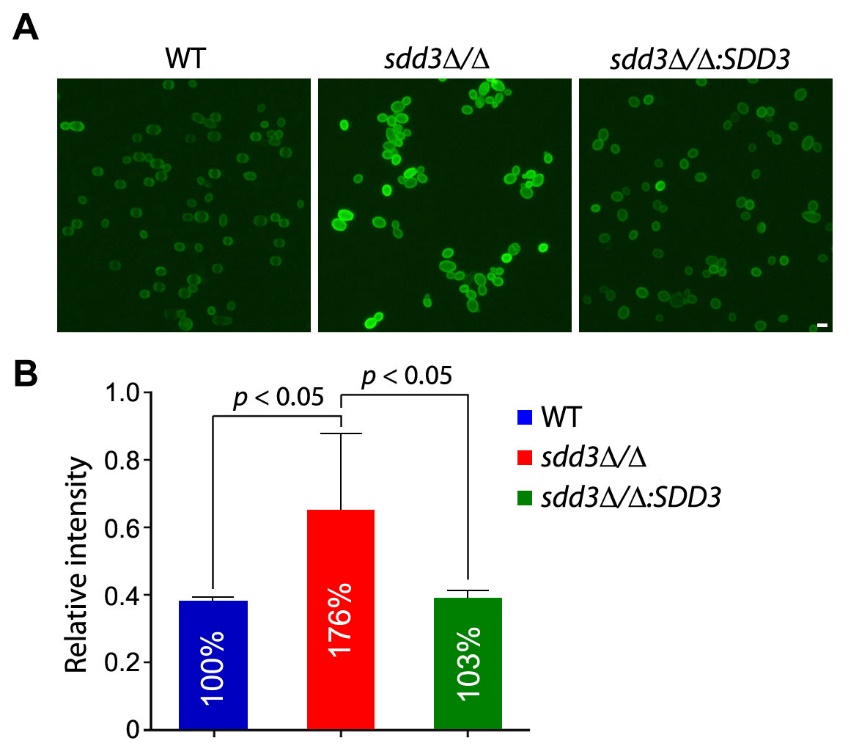
**

**FIG S1** The *sdd3*∆/∆ mutant has elevated level of mannoproteins. (**A**) Examination of mannoprotein levels by ConA-FITC staining. WT, *sdd3*∆/∆, and *sdd3*∆/∆*:SDD3* cells were cultured in GMM, collected, and washed with PBS. Equal number of cells were incubated with 100 µg/ml of ConA-FITC at RT for 45 min in the dark, followed by fixation with 0.3% sodium azide for 1 h. Cells were then examined under a fluorescence microscope, and images were acquired with identical settings. Bar, 5 μm. (**B**) Quantitative analysis of fluorescence intensity of ConA-FITC stained cells. The fluorescence intensities of ≥ 80 cells from each strain were measured using Image J, and the average intensity of one single cell in each group was calculated. The relative fluorescence intensities of *sdd3*∆/∆ and *sdd3*∆/∆*:SDD3* cells against that of WT cells are shown as percentages. The relative intensity values are the means of three independent measurements. Error bars represent SD.

**Table S1:** Yeast strains used in this study

| **Strain** | **Ploidy** | **Genotype** | **Source** |
| --- | --- | --- | --- |
| GZY803 | Haploid | *MTLα his4 ura3Δ::HIS4* | (1) |
| YW02 | Haploid | *MTLα his4 ura3Δ::HIS4 P_Tet-On_-CaPBase::SAT1 arg4::PB[URA3]* | (2) |
| GZY1095 | Haploid | *MTLα his4 ura3Δ::HIS4 bcr1Δ::HIS4* | (3) |
| HIM30 | Haploid | *MTLα his4 ura3Δ::HIS4 P_Tet-On_-CaPBase::SAT1 ARG4 orf19.5210::PB[URA3]* | This study |
| HIM33 | Haploid | *MTLα his4 ura3Δ::HIS4 P_Tet-On_-CaPBase::SAT1 ARG4 PCD1::PB[URA3]* | This study |
| HIM45 | Haploid | *MTLα his4 ura3Δ::HIS4 P_Tet-On_-CaPBase::SAT1 ARG4 ORF19.5210.1::PB[URA3]* | This study |
| HIM48 | Haploid | *MTLα his4 ura3Δ::HIS4 P_Tet-On_-CaPBase::SAT1 ARG4 ORF19.6499::PB[URA3]* | This study |
| HIM101 | Haploid | *MTLα his4 ura3Δ::HIS4 P_Tet-On_-CaPBase::SAT1 ARG4 ORF19.7235::PB[URA3]* | This study |
| HIM118 | Haploid | *MTLα his4 ura3Δ::HIS4 P_Tet-On_-CaPBase::SAT1 ARG4 ORF19.6693::PB[URA3]* | This study |
| HIM139 | Haploid | *MTLα his4 ura3Δ::HIS4 P_Tet-On_-CaPBase::SAT1 ARG4 MNN10::PB[URA3]* | This study |
| HIM170 | Haploid | *MTLα his4 ura3Δ::HIS4 P_Tet-On_-CaPBase::SAT1 ARG4 ORF19.6168::PB[URA3]* | This study |
| HIM249 | Haploid | *MTLα his4 ura3Δ::HIS4 P_Tet-On_-CaPBase::SAT1 ARG4 ORF19.1687::PB[URA3]* | This study |
| HIM250 | Haploid | *MTLα his4 ura3Δ::HIS4 P_Tet-On_-CaPBase::SAT1 ARG4 ORF19.7027::PB[URA3]* | This study |
| HIM295 | Haploid | *MTLα his4 ura3Δ::HIS4 P_Tet-On_-CaPBase::SAT1 ARG4 FGR15::PB[URA3]* | This study |
| HIM313 | Haploid | *MTLα his4 ura3Δ::HIS4 P_Tet-On_-CaPBase::SAT1 ARG4 ORF19.4905::PB[URA3]* | This study |
| HIM444 | Haploid | *MTLα his4 ura3Δ::HIS4 P_Tet-On_-CaPBase::SAT1 ARG4 STI1::PB[URA3]* | This study |
| HIM469 | Haploid | *MTLα his4 ura3Δ::HIS4 P_Tet-On_-CaPBase::SAT1 ARG4 SDH4::PB[URA3]* | This study |
| HIM498 | Haploid | *MTLα his4 ura3Δ::HIS4 P_Tet-On_-CaPBase::SAT1 ARG4 SFL2::PB[URA3]* | This study |
| HIM517 | Haploid | *MTLα his4 ura3Δ::HIS4 P_Tet-On_-CaPBase::SAT1 ARG4 ORF19.1277::PB[URA3]* | This study |
| HIM597 | Haploid | *MTLα his4 ura3Δ::HIS4 P_Tet-On_-CaPBase::SAT1 ARG4 POT1-2::PB[URA3]* | This study |
| SC5314 | Diploid | Wild-type diploid | (4) |
| BWP17 | Diploid | *ura3::imm434/ura3::imm434 his1::hisG/his1::hisG arg4::hisG/arg4::hisG* | (5) |
| BWP17UH | Diploid | (BWP17) *URA3 HIS1* | This study |
| GZY1094 | Diploid | (BWP17) *bcr1*∆*::UFP/bcr1*∆*::HIS1* | This study |
| GZY1387 | Diploid | (BWP17) *sdh4*∆*::HIS1/sdh4*∆*::UFP* | This study |
| GZY1389 | Diploid | (BWP17) *orf19.5210*∆*::HIS1/orf19.5210*∆*::UFP* | This study |
| GZY1398 | Diploid | (BWP17) *sdd3*∆*::HIS1/SDD3* | This study |
| GZY1399 | Diploid | (BWP17) *sdd3*∆*::HIS1/sdd3*∆*::UFP* | This study |
| GZY1412 | Diploid | (BWP17) *sdd3*∆*::HIS1/sdd3*∆*::UFP SDD3-ARG4* | This study |
| GZY1456 | Diploid | (BWP17) *sdd3*∆*::HIS1/sdd3*∆*::FRT* | This study |
| GZY1457 | Diploid | (BWP17) *sdd3*∆*::HIS1/sdd3*∆*::FRT TetOff-Myc-RHO1^G18V^-UTR*-TetR-URA3* | This study |
| GZY1467 | Diploid | (BWP17) *sdd3*∆*::HIS1/sdd3::TetOff-Myc-SDD3-TetR-URA3* | This study |
| GZY1495 | Diploid | (BWP17) *sdd3*∆*::HIS1/sdd3::TetOff-Myc-SDD3-TetR-URA3 BEM2/BEM2-HA-ARG4* | This study |
| GZY1496 | Diploid | (BWP17UH) *BEM2/BEM2-HA-ARG4* | This study |
| GZY1522 | Diploid | (BWP17) *sdd3*∆*::HIS1/sdd3*∆*::FRT TetOff-Myc-CHS8-TetR-URA3* | This study |
| GZY1532 | Diploid | (BWP17) *bem2*∆*::UFP/bem2*∆*::HIS1* | This study |
| GZY1534 | Diploid | (BWP17) *sdd3*∆*::HIS1/sdd3*∆*::FRT bem2*∆*::UFP/bem2*∆*::ARG4* | This study |

1. Hickman MA, Zeng G, Forche A, Hirakawa MP, Abbey D, Harrison BD, Wang YM, Su CH, Bennett RJ, Wang Y, and Berman J. The ‘obligate diploid’ *Candida albicans* forms mating competent haploids. *Nature*. 7, 55-59 (2013).
2. Gao J, Wang H, Li Z, Wong AH, Wang YZ, Guo Y, Lin X, Zeng G, Liu H, Wang Y, and Wang J. *Candida albicans* gains azole resistance by altering sphingolipid composition. *Nat Commun* 9: 4495 (2018).
3. Seneviratne CJ, Zeng G, Truong T, Sze S, Wong W, Samaranayake L, Chan FY, Wang YM, Wang H, Gao J, and Wang Y. New "haploid biofilm model" unravels *IRA2* as a novel regulator of *Candida albicans* biofilm formation. *Sci Rep* srep12433 (2015).
4. Gillum AM, Tsay EY, and Kirsch DR. Isolation of the *Candida albicans* gene for orotidine-5’-phosphate decarboxylase by complementation of *S. cerevisiae ura3* and *E. coli pyrF* mutations. *Mol Gen Genet* 198, 179-182 (1984).
5. Wilson RB, Davis D and Mitchell AP. Rapid hypothesis testing with *Candida albicans* through gene disruption with short homology regions. *J Bacteriol* 181, 1868-1874 (1999).

**Table S2:** Plasmid constructs used in this study

| **Construct** | **Description** |
| --- | --- |
| CIP10U | *C. albicans* integration vector with *URA3* as the selection marker; generated by replacing the *RP10* gene in the vector Clp10^1^ with 700 bp *GAL1* untranslated region (*UTR*) at PstI and MluI sites. |
| CIP10A | *C. albicans* integration vector with *ARG4* as the selection marker; generated by replacing the *URA3* in the vector CIP10U with *ARG4* by MluI and NotI sites. |
| pYGS1211 | BEM2∆::UFP/pKBS; *BEM2* promoter (~800 bp) and terminal (~450 bp) regions were PCR-amplified and cloned into the vector pBKS at KpnI-XhoI and NotI-SacII sites, respectively, to flank the *URA3* flipper^1^ (*UFP*) located between XhoI and NotI. The knock-out cassette was released by KpnI and SacII digestion for transformation to generate *bem2*∆::*UFP*. |
| pYGS1280 | TetOff-Myc-RHO1^G18V^-UTR*-TetR/CIP10U; The mutated *RHO1* gene (*RHO1^G18V^*) was PCR-amplified and cloned into a vector downstream of *TetOff*-*Myc* and upstream of *UTR**-*TetR* by ClaI and PacI. The plasmid was linearized by AscI (within UTR*) for integration. |
| pYGS1428 | SDH4∆::UFP/pKBS; *SDH4* promoter (~500 bp) and terminator (~450 bp) regions were amplified by PCR and cloned into the vector pBKS at KpnI-XhoI and NotI-SacII sites, respectively, to flank *UFP*. The knock-out cassette was released by KpnI and SacII digestion for transformation to generate *sdh4*∆::*UFP*. |
| pYGS1431 | SDH4∆::HIS1/pKBS; *HIS1* was cloned into pYGS1428 at XhoI-NotI to replace *UFP*. The knock-out cassette was released by KpnI and SacII digestion for transformation to generate *sdh4*∆::*HIS1*. |
| pYGS1432 | ORF19.5210∆::UFP/pKBS; *ORF19.5210* promoter (~500 bp) and terminator (~450 bp) regions were amplified by PCR and cloned into the vector pBKS at KpnI-XhoI and NotI-SacII sites, respectively, to flank *UFP*. The knock-out cassette was released by KpnI and SacII digestion for transformation to generate *orf19.5210*∆::*UFP*. |
| pYGS1433 | ORF19.5210::HIS1/pKBS; *HIS1* was cloned into pYGS1432 at XhoI-NotI to replace *UFP*. The knock-out cassette was released by KpnI and SacII digestion for transformation to generate *orf19.5210*::*HIS1*. |
| pYGS1437 | SDD3∆::UFP/pKBS; *SDD3* promoter (~500 bp) and terminator (~450 bp) regions were amplified by PCR and cloned into the vector pBKS at KpnI-XhoI and NotI-SacII sites, respectively, to flank *UFP*. The knock-out cassette was released by KpnI and SacII digestion for transformation to generate *sdd3*∆::*UFP* and *sdd3*∆::*FRT* (by looping out *URA3* from *UFP* via flippase-mediated excision^2^). |
| pYGS1438 | SDD3∆::HIS1/pKBS; *HIS1* was cloned into pYGS1437 at XhoI-NotI to replace *UFP*. The knock-out cassette was released by KpnI and SacII digestion for transformation to generate *sdd3*∆::*HIS1*. |
| pYGS1445 | SDD3/CIP10A; The *SDD3* gene, including its promoter and terminator (527 bp upstream of ATG and 520 bp downstream of STOP), was PCR-amplified and cloned into CIP10A between KpnI and XhoI. The plasmid was linearized by EcoRV (within promoter) for integration. |
| pYGS1483 | TetOff-Myc-SDD3n-TetR/CIP10U; The N terminal region of *SDD3* (1013 bp) was PCR-amplified and cloned into a vector downstream of *TetOff*-*Myc* and upstream of *TetR* by ClaI and PstI. The plasmid was linearized by PacI (within SDD3n) for integration. |
| pYGS1486 | BEM2∆::ARG4/pBKS; *ARG4* was cloned into pYGS1211 at XhoI-NotI to replace *UFP*. The knock-out cassette was released by KpnI and SacII digestion for transformation to generate *bem2*∆::*ARG4*. |
| pYGS1493 | BEM2c-HA/CIP10A; The C terminal region of *BEM2* (1028 bp) was cloned into a vector upstream of *HA* epitope by KpnI and XhoI. The plasmid was linearized by BsaBI (within BEM2c) for integration. |
| pYGS1500 | TetOff-Myc-CHS8n-TetR/CIP10U; N terminal region of *CHS8* (1005 bp) was PCR-amplified and cloned into a vector downstream of *TetOff*-*Myc* and upstream of *TetR* by ClaI and PstI. The plasmid was linearized by SmaI (within CHS8n) for integration. |
| pYGS1507 | BEM2∆HIS1/pBKS; *HIS1* was cloned into pYGS1211 at Xho1-NotI to replace *UFP*. The knock-out cassette was released by KpnI and SacII digestion for transformation to generate *bem2*∆::*HIS1*. |

1. Murad AM, Lee PR, Broadbent ID, Barelle CJ, and Brown AJ. CIp10, an efficient and convenient integrating vector for *Candida albicans*. *Yeast* 16, 325-327 (2000).
2. Morschhauser J, Michel S & Staib P. Sequential gene disruption in *Candida albicans* by FLP-mediated site-specfiic recombination. *Mol Microbiol* 32, 547-556 (1999).

**Table S3:** Genes identified in the genetic screening

| **Gene** | ***PB* location** | **Functions** | **Phenotype** |
| --- | --- | --- | --- |
| *FGR15* | -4 bp | Putative transcription factor with zinc finger DNA-binding motif; lacks an ortholog in *S. cerevisiae*; transposon mutation affects filamentous growth | Defective |
| *MNN10* | -85 bp | α-1,6-mannosyltransferase involved in biosynthesis and organization of cell wall polysaccharides | Defective |
| *PCD1* | -310 bp | Ortholog(s) have 8-oxo-7,8-dihydroguanosine triphosphate pyrophosphatase activity, pyrophosphatase activity, role in DNA repair and peroxisome localization | Enhanced |
| *POT1-2* | 260 bp | Putative peroxisomal 3-ketoacyl CoA thiolase | Defective |
| *SDH4* | 118 bp | Succinate dehydrogenase | Defective |
| *SFL2* | -718 bp | Transcription factor involved in regulation of morphogenesis; regulates transcription in response to carbon dioxide levels; required for filamentous growth, for virulence in RHE model but not in mice | Defective |
| *STI1* | -100 bp | Protein that interacts with Cdc37 and Crk1 in two-hybrid; may be involved in Cdc37 chaperone activity; soluble protein in hyphae | Enhanced |
| *ORF19.1277* | 318 bp | Uncharacterized; protein of unknown function | Defective |
| *ORF19.1687* | -93 bp | Uncharacterized; ortholog of *S. cerevisiae* Prp43, an RNA helicase in the DEAH-box family that functions in both RNA polymerase I and polymerase II transcript metabolism | Defective |
| *ORF19.4905* | 1482 bp | Uncharacterized; putative MFS transporter; regulated by Sef1p and Sfu1p; repressed in a ssr1 null mutant | Defective |
| *ORF19.5210* | -74 bp | Uncharacterized; putative Xbp1 transcriptional repressor; binds to cyclin gene promoters in *S. cerevisiae*; possibly essential, disruptants not obtained by UAU1 method | Enhanced |
| *ORF19.5210.1* | -58 bp | Uncharacterized; protein conserved in *C. dublinensis* | Enhanced |
| *ORF19.6168* | 2175 bp | Uncharacterized; protein of unknown function | Defective |
| *ORF19.6499* | 2973 bp | Uncharacterized; predicted DNA-directed RNA polymerase; role in transcription | Enhanced |
| *ORF19.6693* | 1334 bp | Uncharacterized; has domain(s) with predicted metal ion binding activity | Defective |
| *ORF19.7027* | -1458 bp | Uncharacterized; protein of unknown function | Defective |
| *ORF19.7235* | 2297 bp | Putative protein of unknown function; mutation confers hypersensitivity to amphotericin B | Defective |

**Table S4:** Primers used in this study

| **Primer Name** | **Sequence (5’ to 3’)** | **Purpose** |
| --- | --- | --- |
| PBLf | CGACCGCGTGAGTCAAAATGAC | Inverse PCR |
| PBLr | TCCAAGCGGCGACTGAGATG | Inverse PCR |
| Seq1 | CGCGCTATTTAGAAAGAGAGAG | Inverse PCR |
| SDH4-P01F | CGGGGTACCGATTGATTCCTGGTGTCAAGG | *SDH4* gene deletion |
| SDH4-P02R | CCGCTCGAGTTGATATTGTCTTAATGGTG | *SDH4* gene deletion |
| SDH4- P03F | TGCGGCCGCGGGTCTCAACTCCGTTAGAA | *SDH4* gene deletion |
| SDH4-P04R | TCCCCGCGGTAAACTATATACAACAGCAG | *SDH4* gene deletion |
| SDH4-P05F | CCCAATGATTGATTCGATTT | *SDH4* PCR check |
| SDH4-P06R | AGATCTTGTTCAGGTTTTGA | *SDH4* PCR check |
| ORF19.5210-P01F | CGGGGTACCTTTTGTCCAACAACTTTATT | *ORF19.5210* gene deletion |
| ORF19.5210-P02R | CCGCTCGAGTGGTAAATTTAACTTGACA | *ORF19.5210* gene deletion |
| ORF19.5210- P03F | TGCGGCCGCTTAACACAGTTATATATCCA | *ORF19.5210* gene deletion |
| ORF19.5210-P04R | TCCCCGCGGAATCGTCCAACTTTAATTCG | *ORF19.5210* gene deletion |
| ORF19.5210-P05F | GTATTTCCTCAATCGGTACG | *ORF19.5210* gene deletion |
| ORF19.5210-P06R | TGTTCAATTGGAGGTGATAC | *ORF19.5210* gene deletion |
| SDD3-P01F | CGGGGTACCATATCAATCAGTGAAAGAAT | *SDD3* gene deletion |
| SDD3-P02R | CCGCTCGAGATTCTTTTAGTGTGGTCT | *SDD3* gene deletion |
| SDD3- P03F | TGCGGCCGCATAATGTTGATTTAATTAGA | *SDD3* gene deletion |
| SDD3-P04R | TCCCCGCGGAACAACAAGTTTGTGAAT | *SDD3* gene deletion |
| SDD3-P05F | CTTGGTGTAAAGCTAAAACC | *SDD3* PCR check |
| SDD3-P06R | ACCATATCTCAATGGGTTGC | *SDD3* PCR check |
| SDD3-rescueF | CGGGGTACCGTAAAATCTAAAAATGAACC | *SDD3* rescue |
| SDD3-rescueR | TCCGTCGACACCATATCTCAATGGGTTG | *SDD3 rescue* |
| SDD3n-F | AAACCGATCGATATGCATAGAGAATTTCCGC | *SDD3* tagging |
| SDD3n-R | AAACCGCTGCAGCCTCGAGTGGCTCATCATG | *SDD3* tagging |
| CHS8-OE-F | AAACCGATCGATATGGGTAACTCAAATTTC | *CHS8* overexpression |
| CHS8-OE-R | AAACCGCTGCAGACCATAAGTTTCCAATAAC | *CHS8* overexpression |
| BEM2-P01F | CGGGGTACCAGCTAAGAGGATTGGTCCTAA | *BEM2* gene deletion |
| BEM2-P02R | CCGCTCGAGACCTATACTACAAACTTCTAG | *BEM2* gene deletion |
| BEM2-P03F | AAACCGGCGGCCGCGCCAGCATTAAC | *BEM2* gene deletion |
| BEM2-P04R | AAACCGCCGCGGCATGCTGATGCTTA | *BEM2* gene deletion |
| BEM2-P05F | CTTCCATTAGTGAGTTGA | *BEM2* PCR check |
| BEM2-P06R | CAACACCACTTTGGATAA | *BEM2* PCR check |
| BEM2c-F | AAACCGGGTACCTCAATGATCGGGGATATG | *BEM2* tagging |
| BEM2c-R | AAACCGCTCGAGAAATTGAACTTTAAGCTC | *BEM2* tagging |
| ACT1F | ACTACCATGTTCCCAGGTATTG | qPCR |
| ACT1R | CCACCAATCCAGACAGAGTATT | qPCR |
| CHS2F | AGAGGAAGGTGAGACGAGTT | qPCR |
| CHS2R | CTTGTTGTGGAGGAGGTTCTT | qPCR |
| CHS3F | AACTGGTGTTGATCCTCGTAAA | qPCR |
| CHS3R | GGATCCCTTCGTCTTCTTCTTC | qPCR |
| CHS8F | GTCAATGGAGAAGGTCCGTTAG | qPCR |
| CHS8R | CGTTCATCGTCAAAGTCTGGA | qPCR |

*Underlined regions represent restriction digest sites.
